# In Vitro α-Glucosidase and α-Amylase Inhibition, Cytotoxicity and Free Radical Scavenging Profiling of the 6-Halogeno and Mixed 6,8-Dihalogenated 2-Aryl-4-methyl-1,2-dihydroquinazoline 3-Oxides

**DOI:** 10.3390/antiox12111971

**Published:** 2023-11-06

**Authors:** Nontokozo M. Magwaza, Garland K. More, Samantha Gildenhuys, Malose J. Mphahlele

**Affiliations:** 1 Department of Chemistry, College of Science, Engineering and Technology, University of South Africa, Private Bag X06, Florida 1710, South Africa; magwan@unisa.ac.za; 2 College of Agriculture and Environmental Sciences Laboratories, University of South Africa, Private Bag X06, Florida 1710, South Africa; moregk@unisa.ac.za; 3 Department of Life & Consumer Sciences, College of Agriculture and Environmental Sciences, University of South Africa, Private Bag X06, Florida 1710, South Africa; gildes@unisa.ac.za

**Keywords:** halogenated 1,2-dihydroquinazoline 3-oxides, diabetes, cytotoxicity, antioxidant activity, molecular docking

## Abstract

Series of the 6-bromo/iodo substituted 2-aryl-4-methyl-1,2-dihydroquinazoline-3-oxides and their mixed 6,8-dihalogenated (Br/I and I/Br) derivatives were evaluated for inhibitory properties against α-glucosidase and/or α-amylase activities and for cytotoxicity against breast (MCF-7) and lung (A549) cancer cell lines. The 6-bromo-2-phenyl substituted **3a** and its corresponding 6-bromo-8-iodo-2-phenyl-substituted derivative **3i** exhibited dual activity against α-glucosidase (IC_50_ = 1.08 ± 0.02 μM and 1.01 ± 0.05 μM, respectively) and α-amylase (IC_50_ = 5.33 ± 0.01 μM and 1.18 ± 0.06 μM, respectively) compared to acarbose (IC_50_ = 4.40 ± 0.05 μM and 2.92 ± 0.02 μM, respectively). The 6-iodo-2-(4-fluorophenyl)-substituted derivative **3f**, on the other hand, exhibited strong activity against α-amylase and significant inhibitory effect against α-glucosidase with IC_50_ values of 0.64 ± 0.01 μM and 9.27 ± 0.02 μM, respectively. Compounds **3c**, **3l** and **3p** exhibited the highest activity against α-glucosidase with IC_50_ values of 1.04 ± 0.03, 0.92 ± 0.01 and 0.78 ± 0.05 μM, respectively. Moderate cytotoxicity against the MCF-7 and A549 cell lines was observed for these compounds compared to the anticancer drugs doxorubicin (IC_50_ = 0.25 ± 0.05 μM and 0.36 ± 0.07 μM, respectively) and gefitinib (IC_50_ = 0.19 ± 0.04 μM and 0.25 ± 0.03 μM, respectively), and their IC_50_ values are in the range of 10.38 ± 0.08–25.48 ± 0.08 μM and 11.39 ± 0.12–20.00 ± 0.05 μM, respectively. The test compounds generally exhibited moderate to strong antioxidant capabilities, as demonstrated via robust free radical scavenging activity assays, viz., DPPH and NO. The potential of selected derivatives to inhibit superoxide dismutase (SOD) was also investigated via enzymatic assay in vitro. Molecular docking revealed the N-O moiety as essential to facilitate electrostatic interactions of the test compounds with the protein residues in the active site of α-glucosidase and α-amylase. The presence of bromine and/or iodine atoms resulted in increased hydrophobic (alkyl and/or π-alkyl) interactions and therefore increased inhibitory effect against both enzymes.

## 1. Introduction

Type 2 diabetes mellitus (T2DM) is a non-communicable disease of increasing concern to society and national governments globally due to its high mortality rate and lifestyle changes, and treatment with natural resources is not enough to completely eradicate this metabolic disorder [1]. This disease is characterized by sustained high levels of glucose in the blood, known as hyperglycemia, caused by impaired insulin secretion by the pancreatic β cells and/or insulin resistance in target tissues [2]. The inhibition of α-amylase and α-glucosidase located in the brush border of the intestine represents an important strategy in the administration of non-insulin-dependent diabetes to control postprandial blood glucose levels in T2DM patients [3]. Preclinical and clinical studies highlight the fact that T2DM is a risk factor for cancer [4], and inhibition of α-glucosidase has been found to have positive effects in the treatment of cancer [5]. Oxidative stress is implicated in the pathogenesis and progression of T2DM due to disruption of the pro-oxidant/antioxidant balance, which causes insulin resistance [6,7]. Normalization of blood glucose levels, especially postprandial hyperglycemia, is important to prevent complications of T2DM such as stroke, cardiovascular heart diseases and cancer due to oxidative stress [8]. A contemporary strategy for the treatment of this multifactorial metabolic disorder involves targeting two or more biochemical pathways implicated in its pathogenesis and/or progression along with oxidative stress.

**Figure 1:**
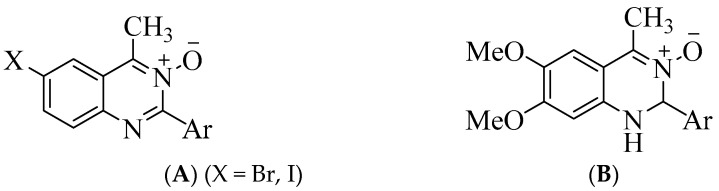
Chemical structures of quinazoline 3-oxides (**A**) and 1,2-dihydroquinazoline 3-oxides (**B**).

Heterocyclic *N*-oxides have emerged as promising agents against a number of diseases and disorders as anticancer, antibacterial, antihypertensive, antiparasitic, anti-HIV, anti-inflammatory, herbicidal and neuroprotective agents [9,10]. X-ray crystallographic data suggest that the N^+^–O^−^ group forms critical hydrogen bonding networks with amino acids in the active site of enzymes, resulting in conformational changes that lead to allosteric modulation of the enzyme function [10]. The biochemical activity of *N*-oxides is also attributed to the donor properties of the N^+^–O^−^ group and its spatial accessibility, which facilitate complexation with the metalloporphyrins in living organisms [11]. Considerable effort has been devoted to the synthesis of quinazoline 3-oxides due to their pharmacological activities as bronchodilators, cardiotonics and fungicides [12,13]. A series of the 6-halogeno (X = Br, I) substituted 2-aryl-4-methylquinazoline 3-oxide derivatives **A** (Figure 1) were synthesized and, in turn, evaluated via enzymatic assays in vitro for inhibitory effect against cyclooxygenase-1/2 (COX-1/2) and lipoxygenase-5 (LOX-5) activities [14]. Structure–activity relationship analysis and molecular docking studies suggested that the presence of a halogen atom at the C-6 position and a 2-aryl group of these heteroaromatic *N*-oxides enhances the inhibitory effect against COX-2. Unlike the quinazoline 3-oxides, which have been extensively evaluated for biological properties [15], corresponding data for their 1,2-dihydroquinazoline 3-oxide precursors are considerably less documented [16,17]. The 1,2-dihydroquinazoline 3-oxides have previously been evaluated for cytotoxicity against the human promyelocytic leukaemia HL-60 and lymphoblastic leukaemia NALM-6 cell lines [16]. A series of the 2-aryl-l,2-dihydro-6,7-dimethoxy-4-methylquinazoline 3-oxides **B**, on the other hand, was found to exhibit cardiotonic and blood pressure-lowering activity in an open-chest anesthetized dog model [17]. The most potent 1,2-dihydroquinazoline 3-oxide (Ar = Ph) exhibited good in vivo cardiotonic activity, and its potency was found to depend on the oxidation state of the heterocyclic ring. Dehydrogenation of **B** to the quinazoline 3-oxide or dehydrogenation-deoxygenation of the most active derivative (Ar = Ph) to the corresponding 6,7-dimethoxy-4-methyl-2-phenylquinazoline resulted in a loss of activity for both these fully aromatic derivatives. This led the authors to infer that if the fully aromatized derivatives were possible metabolites of the active 1,2-dihydroquinazoline 3-oxide moiety, then these aromatic derivatives would not lend activity to the in vivo profile.

A significant number of drugs on the market and drug candidates in clinical development to yield advanced agrochemicals and pharmaceuticals are halogenated derivatives [18,19,20]. There has also been a rise in the number of commercial drugs containing mixed halogens such as one or more fluorine, chlorine, bromine or iodine atoms in addition to one or more further halogen atom/s [21]. Halogenation holds the promise of effective drug design by facilitating drug molecules’ crossing of biological barriers, filling small hydrophobic pockets present in protein targets, prolonging lifetime and having the quality of easy adsorption [22]. Moreover, bromine and iodine contain a region with positive charge (σ-hole), which is responsible for the atom’s halogen bonding (XB) formation and hydrogen bonding to lone pair acceptors to stabilize drug–receptor interactions [23,24]. Although a bromine atom is frequently present as a substituent in halogenated compounds isolated from marine organisms [25], only 4% of Food and Drug Administration (FDA)-approved drugs contain this element [19]. The more labile carbon-iodine bonds, on the other hand, are commonly exploited for the synthesis of novel organic compounds and bioactive agents. However, this halogen atom is the rarest (1%) in medicine in the form of drugs or diagnostic aids [26]. The C(sp^2^)–I bond has been found to be more resistant to deiodination in vivo than the C(sp)–I or C(sp^3^)–C(sp)–I bonds [27]. Moreover, a single iodine moiety was found to facilitate ligand binding to the Y220C mutant of the p53 tumor suppressor via energetically favorable halogen bonding compared to di-iodinated derivatives [28]. With the aforementioned considerations in mind, we decided to synthesize the 2-aryl-4-methyl-1,2-dihydroquinazoline 3-oxides with bromine or iodine substituted at the C-6 position and their corresponding mixed 6,8-dihalogenated (Br, I or I, Br) derivatives for further studies of biological activity as potential antidiabetic agents. The molecular structures of these compounds were evaluated using a combination of NMR, IR and HR-MS spectroscopic techniques. An X-ray structure of a representative compound is also presented. The compounds were evaluated via enzymatic assays in vitro for inhibitory effect against α-glucosidase and α-amylase activities. The compounds were also screened for antigrowth effect against the human breast (MCF-7) and lung (A549) cancer cell lines and for cytotoxicity against the human embryonic kidney (HEK293-T) cell line. The potential of the test compounds as antioxidants was evaluated using the 2,2-diphenyl-1-picrylhydrazyl (DPPH) and nitric oxide (NO) free radical scavenging assays. Derivatives that exhibited significant or increased NO radical scavenging activity were also evaluated via enzymatic assay in vitro for the potential to inhibit nitric oxide synthase (NOS) or superoxide dismutase (SOD) activity. The most active compounds against α-glucosidase and/or α-amylase were selected for a molecular docking study against the test enzymes to correlate the results of in silico and in vitro studies. Moreover, computational methods were also employed to predict the absorption, distribution, metabolism and excretion (ADME) properties of these 2-aryl-1,2-dihydroquinazoline 3-oxide derivatives.

## 2. Materials and Methods

### 2.1. Materials and Instrumentation

All starting materials and solvents used in this study were procured from Merck in South Africa (Modderfontein, Johannesburg, South Africa) and used without further purification. The melting point (mp.) values of the prepared compounds were recorded on a Thermocouple digital melting point apparatus (Mettler Toledo LLC, Columbus, OH, USA). Merck kieselgel 60 (0.063–0.200 mm) (Merck KGaA, Frankfurt, Germany) was used as a stationary phase for column chromatography. The infrared (IR) spectra were recorded using the thin-film method on a Bruker VERTEX 70 FT-IR Spectrometer (Bruker Optics, Billerica, MA, USA) equipped with an ATR (diamond attenuated total reflectance) accessory. Merck kieselgel 60 (0.063–0.200 mm) (Merck KGaA, Frankfurt, Germany) was used as a stationary phase for column chromatography. The ^1^H-NMR and ^13^C-NMR spectra were recorded as deuterated chloroform (CDCl_3_) or dimethyl sulfoxide (DMSO-*d*_6_) solutions using Agilent 500 MHz NMR spectrometer (Agilent Technologies, Oxford, UK) operating at 500 MHz and 125 MHz for ^1^H and ^13^C, respectively. The chemical shifts are quoted relative to tetramethylsilane (TMS) used as an internal reference standard (δ = 0.00 ppm) or to residual protonated solvent. HR-MS analysis was performed on a QTOF Bruker Impact II (Bruker Daltonics GmbH Fahrensheitstr. 4, Bremen, Germany) in positive ESI mode. The ion source temperature was set to 550 °C, with the IS voltage set to 5500 V. The analytical data of compounds **2a–d** and **3a**–**p** and the corresponding nuclear magnetic resonance (NMR) spectra (^1^H- and ^13^C-NMR) have been included as Figure S1 in the Supplementary Materials.

### 2.2. Typical Procedure for the Oximation of ***1a**–**d***

A stirred mixture of **1a** (2.00 g, 14.09 mmol), hydroxylamine hydrochloride (1.96 g, 28.18 mmol) and pyridine (2.23 g, 28.18 mmol) in absolute ethanol (20 mL) was heated at 80 °C for 5 h. Upon completion of the reaction (thin-layer chromatography monitoring), the mixture was allowed to cool and then quenched with ice-cold water. The resultant precipitate was filtered and recrystallized from ethanol to afford a pure product. Compounds **2a**–**d** were prepared in this fashion.

### 2.3. Typical Procedure for the Synthesis of ***3a**–**p***

A mixture of **2a** (0.50 g, 1.97 mmol) and *p*-TsOH (0.03 g, 0.19 mmol) in ethanol (50 mL) was stirred at RT for 10 min. before the addition of benzaldehyde (0.21 g, 1.98 mmol). The reaction was then stirred at RT for 30 min. and then concentrated under reduced pressure using a rotary evaporator. The residue was dissolved in dichloromethane (50 mL), and the organic solution was washed with water (3 × 10 mL) and then dried over anhydr. MgSO_4_. The salt was filtered off, and the solvent was evaporated under reduced pressure on a rotary evaporator. The residue was purified using column chromatography on silica gel using a 4:1 toluene-ethyl acetate mixture (*v*/*v*) as an eluent. Compounds **3a**–**p** were prepared in 50–96% yield in this fashion.

### 2.4. Inhibition of α-Glucosidase and α-Amylase Activities by ***3a**–**p***

#### 2.4.1. Inhibition of α-Glucosidase

α-Glucosidase type 1 from baker’s yeast (G5003), *p*-nitrophenyl-α-D-glucopyranoside (PNP-G, N1377) and acarbose (A8980) were purchased from Sigma Aldrich (Pty) Ltd. (Johannesburg, South Africa). The assay was conducted in triplicate following a method described in our previous study [29]. The stock solutions of compounds **3a**–**p** (1000 µM) and acarbose were prepared in DMSO, followed by dilution with 100 mM phosphate buffer to obtain the concentrations of 1, 5, 10, 25 and 50 µM. Acarbose was used as a positive control at the same concentrations. The enzyme solution (0.48 u/mL α-glucosidase, 17 µL), phosphate buffer (100 mM, pH 6.8; 50 µL) and test sample in DMSO (17 µL) were incubated at 37 °C for 10 min. After pre-incubation, 17 µL of 2 mM PNP-G was added. The absorbances were recorded in a 96-well microplate at 400 nm using an Elisa plate reader (Varioskan Flash, ThermoFischer Scientific, Vantaa, Finland). The average values obtained from the readings were used to determine the IC_50_ and standard deviation values using a GraphPad Prism software (Version 8, GraphPad Software Inc., San Diego, CA, USA). The average values obtained from the absorbance readings were used to determine the IC_50_ and standard deviation values in GraphPad prism from nonlinear regression dose–response curves.

#### 2.4.2. Inhibition of α-Amylase by **3a**–**p**

The α-amylase assay was performed in triplicate, using a 96-well plate following the procedure recommended by the manufacturer as outlined in the α-amylase inhibitor Screening Kit (Catalogue No. ab283391; Abcam). The stock solution (1000 µM) of the test compounds and acarbose were prepared in DMSO and further diluted with α-amylase assay buffer to obtain final concentrations of 1, 5, 10, 25 and 50 µM. Test samples of 50 µL each at different concentrations and acarbose were incubated with 50 µL α-amylase solution at room temperature for 10 min. After pre-incubation, 50 µL of the substrate was added, and absorbance was identified at a wavelength of 405 nm using an Elisa plate reader (Varioskan Flash, ThermoFisher Scientific, Vantaa, Finland). The average values obtained from the absorbance readings were used to determine the IC_50_ and standard deviation values in GraphPad prism from nonlinear regression dose–response curves.

### 2.5. In Vitro Cytotoxicity of ***3a**–**p*** against the MCF-7, A549 and HEK293-T Cell Lines

Cytotoxicity was measured in breast cancer (MCF-7), lung cancer (A549) and the HEK293-T cell lines (Cellonex Separation Scientific SA (Pty) Ltd. (Roodepoort, Johannesburg, South Africa) using the standard 3-[4,5-dimethylthiazol-2-yl]-2,5-diphenyltetrazolium bromide (MTT) assay [15] against doxorubicin and gefitinib as positive controls. Cells were cultured in sterile Dulbecco’s Minimal Essential Medium (DMEM, Gibco) supplemented with 10% fetal bovine serum (FBS) and 1% penicillin-streptomycin solution. After 24 h incubation at 37 °C in 5% CO_2,_ when the cells reached 80% confluency, they were harvested using 2% Trypsin-EDTA solution and centrifuged for 5 min. at 3000 RCM and re-suspended in DMEM. Cell counting was carried out using a handheld automated cell counter (Scapter 3.0™, Merck, Burlington, MA, USA) and seeded (1 × 10^4^ cells/well) into a 96-well plate. After 24 h, the cells were treated with different concentrations (1, 5, 10, 25 and 50 µM) of the test compounds and reference standards prepared in DMEM. The MTT solution (20 μL) prepared in PBS (5 mg/mL) was added to all the wells, and the plates were incubated for 4 h, followed by 1 h incubation with 100 µL of dimethyl sulfoxide (DMSO) to dissolve the formazan crystals. Untreated cells and 5% DMSO were included in the experiment as negative controls. All experiments were performed in triplicate. The plates were read at 570 nm at a reference wavelength of 630 nm using an ELISA plate reader (Varioskan Flash, ThermoFisher Scientific, Vantaa, Finland). The percentage of cell viability was calculated using the following formula,

$$\mathrm{Cell}\;\mathrm{viability}\;\left(\%\right)=\frac{\mathrm{At}}{\mathrm{Ab}}\times 100$$

where At represents the absorbance value of test compound and Ab the absorbance value of the blank. The IC_50_ values, i.e., the concentration of the drug that inhibits 50% of cells, were obtained from dose–response curves, and comparisons were assessed via a one-way ANOVA performed using Prism 5.0 (GraphPad Software Inc., San Diego, CA, USA). The data were expressed as means ± SD.

### 2.6. Free Radical Scavenging Assays of ***3a**–**p***

#### 2.6.1. The 2,2-Diphenyl-1-picrylhydrazyl (DPPH) Radical Scavenging Assay

The DPPH radical scavenging assay was conducted following the procedure described in our previous study [15]. The test compounds and ascorbic acid at various concentrations (ranging from 0 µM to 50 µM) in DMSO were mixed with a solution of DPPH (0.20 mM) in methanol and incubated in the dark for 45 min. Five absorbance readings were recorded at 512 nm using an Elisa plate reader (Varioskan Flash, ThermoFisher Scientific, Vantaa, Finland). The experiment was carried out in triplicate, and averaged values from the absorbance readings were used to determine the IC_50_ and standard deviation values in GraphPad prism from nonlinear regression dose–response curves.

#### 2.6.2. NO Free Radical Scavenging Assay

The assay was conducted following the method described in our previous study [15]. A mixture of 10 mM sodium nitroprusside (20 µL), phosphate buffer (5 µL) and the test compound (5 µL) were incubated at 25 °C in a 96-well plate for 2.5 h. After incubation, 20 µL of Griess reagent (1.00 g of sulfanilic acid + 0.10 g naphthylethylene diamine dihydrochloride) was added to the previous mixture and allowed to stand for 30 min. The absorbance of the color developed during diazotization of nitrite with sulfanilamide and its subsequent coupling with napthylethylenediamine hydrochloride was observed at 550 nm using an Elisa plate reader (Varioskan Flash, ThermoFisher Scientific, Vantaa, Finland). Each experiment was performed in triplicate, and the average was taken. The average obtained from the absorbance readings was used to calculate the IC_50_ values in GraphPad prism from nonlinear regression dose–response curves.

#### 2.6.3. Assay on **3a**, **3c**, **3f**, **3i**, **3l**, **3n** and **3p** for Inhibitory Activity against NO Generated from NOS

A nitric oxide synthase (NOS) assay was performed following the procedure recommended by the manufacturer as outlined in the screening kit (Catalogue No. EINO-100; EnzyChrom). Various concentrations (1, 5, 10, 25 and 50 µM) of the compounds **3a**, **3c**, **3f**, **3i**, **3l**, **3n** and **3p** and the reference standard, ascorbic acid, were prepared. The enzyme solution (12.5 U/mL NOS, 10 µL), assay buffer (25 µL) and test samples (5 µL) were incubated at room temperature for 15 min. After pre-incubation, 10 µL of reaction mix was added and incubated at 37 °C for 60 min. A 200 µL quantity of NO detection reagent was added, the detection reaction was run at 37 °C for 60 min, and absorbance was determined at a wavelength of 540 nm using an Elisa plate reader (Varioskan Flash, ThermoFisher Scientific, Vantaa, Finland). The average of three readings was used to calculate the NOS percentage inhibition using the following formula:

$$\%\;\mathrm{Inhibition}=\left(1-\frac{\Delta \mathrm{OD}\;\mathrm{Test}\;\mathrm{Compd}}{\Delta \mathrm{OD}\;\mathrm{No}\;\mathrm{Inhibitor}\;}\right)\times 100\%$$

The IC_50_ values and standard deviation values were determined from nonlinear regression dose–response curve and compared with the results in GraphPad prism.

#### 2.6.4. Kinetic Study on Inhibitory Activity of NOS by **3i** and Diphenyleneiodonium (DPI)

A nitric oxide synthase (NOS) assay was performed following the procedure recommended by the manufacturer as outlined in the screening kit (Catalogue No. EINO-100; EnzyChrom). Various concentrations (1, 2.5, 4.0, 5.5, 7.0, 8.5 and 10.0 µM) of the compounds **3i** and the reference standard, DPI, were prepared. The enzyme solution (12.5 U/mL NOS, 10 uL), assay buffer (25 µL) and test samples (5 µL) were incubated at room temperature for 15 min. After pre-incubation, 10 µL of reaction mix was added and incubated at 37 °C for 60 min. A 200 µL quantity of NO detection reagent was added, the detection reaction was run at 37 °C for 60 min, and absorbance was determined at a wavelength of 540 nm using an Elisa plate reader (Varioskan Flash, ThermoFisher Scientific, Vantaa, Finland). The average of three readings was used to calculate the NOS percentage inhibition using the formula in Section 2.6.3

### 2.7. Superoxide Dismutase (SOD) Inhibitory Assay on ***3a**, **3c**, **3f**, **3i**, **3l**, **3n*** and ***3p***

The antioxidant activity of the compounds **3a**, **3c**, **3f**, **3i**, **3l**, **3n** and **3p** against superoxide radicals was assessed via enzymatic assay in vitro using the SOD assay kit-WST according to the manufacturer’s instructions (Sigma-Aldrich^®^). The reaction mixtures in the SOD kit were combined with 20 μL of working solution of the samples in a 96-well plate. Plates were gently shaken and then incubated at 25 °C for 30 min. The reference positive control, diphenyleneiodonium (DPI), and the compounds were tested at 01, 5, 10, 25 and 50 µM in duplicates. The inhibition activity of SOD on the reaction of xanthine oxide generated the superoxide with a tetrazolium salt, which was then determined by measuring the absorbance of the mixtures at a wavelength of 450 nm using an Elisa plate reader (Varioskan Flash, ThermoFisher Scientific, Vantaa, Finland). The SOD activity values obtained used to calculate the percentage of inhibition.

$$\%\;\mathrm{SOD}\;\mathrm{activity}=\left(\frac{\mathrm{ODx}-\;\mathrm{ODy}}{\mathrm{ODx}\;}\right)\times 100\%$$

where ODx is the absorbance of the blank (untreated SOD) and ODy is the absorbance of the sample (treated SOD). All tests were performed in duplicate. Means of samples were used to calculate 50% inhibition of SOD (IC_50_ value) using a nonlinear regression algorithm in GraphPad Prism software (Version 8).

### 2.8. Molecular Docking of Representative Compounds ***3a**, **3c**, **3f**, **3i**, **3l*** and ***3p***

#### 2.8.1. Molecular Docking into α-Glucosidase

Discovery Studio version v20.1.0.19295 was used to dock the compounds to the human α-glucosidase enzyme (PDB code 5NN8). The protein was prepared using default settings for the “prepare protein” step, except that bound ligands were removed. The defined ligand binding site selected aligned with the position of the bound ligand from the original structure, and the x, y, z coordinates were −16.5323, −38.2485, 96.4584 with a radius of 12. Compounds were constructed using the small molecules tools and prepared using the default settings for the “prepare ligand” protocol. Acarbose, which was also the co-crystallized ligand, was also prepared. Docking was conducted for acarbose and the compounds using C-DOCKER. Then the top 5 poses from the docking were subjected to binding energy calculation protocols. The default settings were used, but the active site residues were allowed to flex and the solvent model Generalized Born with Molecular Volume was selected. The pose with the top binding energy and no unfavorable interactions was selected. 

#### 2.8.2. Molecular Docking into α-Amylase

Molecular docking was conducted using Discovery Studio version v20.1.0.19295. Compounds were drawn using the small molecule tools; acarbose was obtained from Pubchem but was also the co-crystallized ligand. Both structures were used to optimize docking parameters. Compounds and acarbose were prepared using the prepare ligands default settings. The protein PDB structure (code 3BAJ) was downloaded and prepared using the prepare protein default settings, except that co-crystalized ligands were set to be removed. Once the protein was prepared, the binding sites were identified from receptor cavities, and the site that aligned with the co-crystalized ligand position was selected for docking (x, y, z coordinates 10.5622, 16.2775, 37.4422 with radius 12.1). CDOCKER was used to conduct docking, and then the top 5 poses from the docking were subjected to binding energy calculation protocols. The default settings were used, but the active site residues were allowed to flex, and the solvent model Generalized Born with Molecular Volume was selected. The pose with the top binding energy and no unfavorable interactions was selected.

### 2.9. Drug-Likeness Estimation of Selected Compounds ***3a**–**p***

Compounds in the SMILES format were entered into the online tool Swiss ADME (http://www.swissadme.ch/index.php, accessed on 20 September 2023) [30], and the data were collected. 

## 3. Results and Discussion

### 3.1. Chemistry

**Scheme 1:**
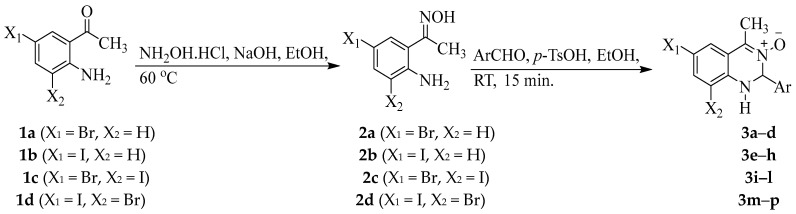
Synthesis of the 2-aryl-4-methyl-1,2-dihydroquinazoline 3-oxides **3a**–**p**.

The 2-aryl-4-methyl-1,2-dihydroquinazoline 3-oxides were prepared in two steps following a method previously employed for the synthesis of the A-ring unsubstituted analogues [31], as shown in Scheme 1. The designation of substituents and percentage yields of the isolated products are represented in Table 1. Oximation of the halogenated 2-aminoacetophenones **1a**–**d** was achieved with hydroxylamine hydrochloride under basic conditions in ethanol at 60 °C to afford derivatives **2a**–**d**. The ^1^H-NMR spectra of these oxime derivatives revealed the presence of a singlet around δ = 6.00 ppm, which corresponds to the hydroxyl proton. These oximes were, in turn, condensed with various benzaldehyde derivatives in the presence of *p*-toluenesulfonic acid (*p-*TsOH) in ethanol at room temperature (RT) to furnish products characterized using a combination of spectroscopic (NMR, IR and HR-MS) techniques as the corresponding 2-aryl-4-methyl-1,2-dihydroquinazoline 3-oxides **3a**–**p**. The proton NMR spectra of these compounds revealed the presence of a set of signals in the region δ 5.18–5.62 ppm (br s) and δ 5.78–6.38 ppm for the amine (NH) and methine (-CH-) protons, respectively. Only the resonance for the methine proton of the 6-bromo-8-iodo-substituted derivatives **3i**–**l** resonated as a doublet around δ = 6.22 ppm with coupling constant value, *J* = 3.5 Hz. The resonance of the methine proton for the other analogues showed the effects of quadrupole broadening due to interaction with the nitrogen atom. This effect usually reduces the accuracy in the measurement of the spin–spin coupling constants of the protons in direct proximity to the nitrogen [32]. Their proton and carbon-13 NMR spectra revealed the presence of an increased number of aromatic proton and carbon signals consistent with the assigned structures. The π-type O–N back-donation is known to increase electronic density at its neighboring carbon atoms. The carbon-13 NMR signal of the methine carbon (C-2) and C-4 resonated around δ = 79.3 ppm and 140.0 ppm, respectively. The stereochemical understanding of the compounds results in important data regarding the most stable conformer of equilibrium, which in turn is the most likely structure exhibiting biological activity in vivo [33]. The lack of heteronuclear and/or vicinal *^3^J*_HH_ scalar coupling constants for the test compounds made it difficult to easily determine the conformation of the heterocyclic ring in the solution phase by means of NMR spectrometric techniques. Moreover, NMR spectrometric data cannot easily distinguish between an N-O inner ring and an N-O coordination bond in the structure. Single-crystal X-ray diffraction (XRD) has established itself as the most accurate method to determine the structure of *N*-oxides and their conformation even more so. Knowledge of conformational preference in the solid state, on the other hand, is very important in drug design and the pharmaceutical industry, as a crystal structure is directly related to the bioavailability of a drug molecule [34]. Moreover, it has been proven that the conformation of medium-sized molecules in solution resembles their solid-state conformation [35].

**Figure 2:**
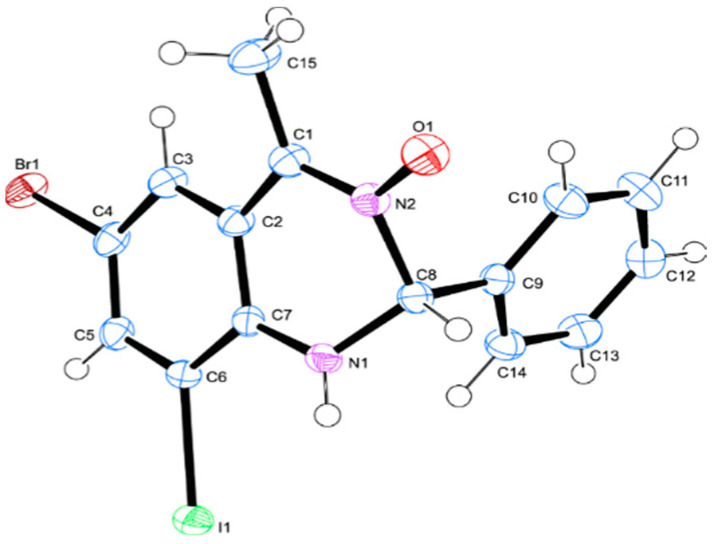
ORTEP diagram of compound **3i** showing the atom numbering scheme used for discussion in the crystallographic section with displacement ellipsoids drawn at the 50% probability level.

We were able to obtain crystals of **3i** suitable for XRD analysis via slow evaporation of acetonitrile. Experimental details of the X-ray analyses for **3i** are provided in Table S1 of the Supplementary Materials. The compound crystallized in the monoclinic *P*21/n space group containing a single molecule in the asymmetric unit is shown in Figure 2. The pyrimidine ring adopts a skew-boat conformation, which is assumed to be adopted by all members of a given series. There is intermolecular hydrogen bonding (N(1)-H(1)⋯O(1)) interaction between the amine proton of one molecule and the oxygen atom of the next partner, with bond distance of 2.879(3) Å and bond angle of 143(3)°. The N(2)–O(1) bond length of 1.305(2) Å is slightly greater than the mean d(N^+^–O^−^) of the acyclic imine *N*-oxides (1.301 Å) and approaching that of the aromatic *N*-oxides (1.318 Å) [36]. This observation reflects the possible involvement of the nitrogen atom in π-electron delocalization with the fused benzo ring.

We envisaged that the presence of a potential hydrogen bond donating NH and accepting N-O functional heads, as well as potential halogen bonding Br and/or I on the scaffold of these heterocycle *N*-oxides, would stabilize the interactions of drug molecules with the receptors. To test our hypothesis and provide some proof of concept, we evaluated the prepared *N*-oxides for inhibitory effect in vitro via enzymatic assays against α-glucosidase and α-amylase activities to correlate between both structural variations and biological activity. Their potential as cytotoxic agents was evaluated in vitro against the MCF-7 and A549 cancer cell lines and the HEK293-T cell line. The compounds were also evaluated for free radical scavenging properties.

### 3.2. Bio-Evaluation of Compounds ***3a**–**p***

The full series comprised the 6-bromo (**3a**–**d**), 6-iodo (**3e**–**h**), 6-bromo-8-iodo (**3i**–**l**) and 6-iodo-8-bromo (**3m**–**p**) sub-series. Some speculation about the structure–activity relationship (SAR) has been made by considering the substitution pattern of the halogen atoms on the fused benzo ring and the type and nature of the substituent on the 2-phenyl ring.

#### Inhibition of α-Glucosidase and α-Amylase and Cytotoxicity Studies of **3a**–**p**

The inhibitory activities of **3a**–**p** were evaluated using α-glucosidase from the *Saccharomyces cerevisiae* (baker’s yeast) and the human α-amylase. All the compounds and the reference standard (acarbose) were evaluated for inhibitory activity against these carbohydrate-hydrolyzing enzymes in the concentration range consisting of 1, 5, 10, 25 and 50 µM. The half-maximal inhibitory concentration (IC_50_) values determined in this concentration range with respect to the enzyme tested are summarized in Table 2 below. A combination of the moderately π-electron-delocalizing lipophilic bromine atom on the fused benzo ring and a 2-phenyl ring in **3a** resulted in increased activity against α-glucosidase and a significant inhibitory effect against α-amylase compared to acarbose (IC_50_ = 4.40 ± 0.04 µM and 2.92 ± 0.02 µM, respectively) with IC_50_ values of 1.08 ± 0.02 µM and 5.33 ± 0.01 µM for this compound against these enzymes, respectively. The 2-(4-fluorophenyl)-substituted derivative **3b** exhibited moderate inhibitory activity against α-glucosidase, with an IC_50_ value of 7.47 ± 0.05 µM, and significantly reduced activity against α-amylase (IC_50_ = 36.35 ± 0.01 µM). However, a combination of the 6-bromo and 2-(4-chlorophenyl) group on the scaffold of **3c** resulted in the highest activity against α-glucosidase (IC_50_ = 0.92 ± 0.01 µM) within the category of **3a**–**d**. The high potency of **3c** might be due to the electron-withdrawing inductive effect of the chlorine atom. The effect is known to increase the lipophilicity of the Cl substituent locally and, in turn, a strong increase of the overall lipophilicity of the whole molecule leading to higher adsorption to proteins such as glucosidase and other enzymes [37]. A strong π-electron-delocalizing 4-methoxyphenyl group in **3d** resulted in significantly reduced activity against both enzymes. Moderate inhibitory effect against α-glucosidase and significantly reduced activity against α-amylase were observed for **3e** substituted with iodine and a phenyl group at the C-6 and C-2 positions of the 1,2-dihydroquinazoline 3-oxide framework, respectively. A combination of iodine at the C-6 position and a moderately π-electron-delocalizing 4-fluorophenyl substituent at the C-2 position of **3f**, on the other hand, resulted in the highest activity against α-amylase (IC_50_ = 0.64 ± 0.01 μM) among the test compounds and more so than acarbose. The other derivatives within the **3e**–**h** series were found to be less active against α-amylase. A combination of a 2-phenyl group and the 6-bromo-8-iodo substituents on the fused benzo ring of **3i** resulted in increased inhibitory effect against α-glucosidase and α-amylase with IC_50_ values of 1.01 ± 0.05 μM and 1.18 ± 0.06 μM, respectively. This compound exhibited higher activity against both enzymes compared to **3a**-substituted with only bromine atom on the fused benzo ring. Relatively weak activity against α-glucosidase and α-amylase was observed for the 2-(4-fluorophenyl) derivative **3j** with IC_50_ values of 19.69 ± 0.01 μM and 17.48 ± 0.03 μM, respectively. The analogous 2-(4-chlorophenyl) derivative **3k** exhibited moderate activity against α-glucosidase (IC_50_ = 13.47 ± 0.04 μM), but significant inhibitory effect against α-amylase with an IC_50_ value of 4.46 ± 0.02 μM. The presence of a strongly π-electron-delocalizing 2-(4-methoxyphenyl) group on the scaffold of **3l** resulted in an increased inhibitory effect against α-glucosidase with an IC_50_ value of 1.04 ± 0.03 μM. However, this compound exhibited significantly reduced activity against α-amylase with an IC_50_ value of 54.18 ± 0.01 μM. The 8-bromo-6-iodo-2-phenyl-substituted derivative **3m** was found to be moderately inhibiting against α-glucosidase (IC_50_ = 9.14 ± 0.03 μM) and α-amylase (IC_50_ = 16.73 ± 0.01 μM) and to be less active compared to the isomeric **3i**. Derivative **3n** exhibited significantly reduced activity against α-glucosidase (IC_50_ = 43.23 ± 0.05 μM) among all the test compounds, but it exhibited a significant inhibitory effect against α-amylase (IC_50_ = 4.71 ± 0.01 μM) within the series **3m**–**p**. The 2-(4-chlorophenyl) derivative **3o** exhibited significant activity against α-glucosidase and reduced inhibitory effect against α-amylase, with IC_50_ values of 7.07 ± 0.04 μM and 49.18 ± 0.01 μM, respectively. The presence of the 2-(4-methoxyphenyl) group in **3p** resulted in increased activity against α-glucosidase (IC_50_ = 0.78 ± 0.05 μM) compared to the isomeric **3l**. Although **3p** was the most active against α-glucosidase, this compound was the least active against α-amylase among the test compounds, with an IC_50_ value of 73.66 ± 0.02 μM. Compounds **3f**, **3k** and **3n,** with their increased activity against α-amylase, will probably slow down the digestion and assimilation of starch at the early stages of digestion, resulting in a substantial delay in postprandial hyperglycemia. The 2-phenyl-substituted derivatives **3a** and **3i**, with the potential to serve as dual inhibitors of α-glucosidase and α-amylase activities, would suppress carbohydrate digestion, delay glucose uptake and result in reduced blood sugar levels. Compounds **3c**, **3e**, **3l** and **3p**, on the other hand, exhibited selectivity against α-glucosidase activity. The inhibition of α-glucosidase represents an important strategy in the administration of non-insulin-dependent diabetes to control postprandial blood glucose levels in T2DM patients. Moreover, inhibition of α-glucosidase has been found to have positive effects in the treatment of non-vascular pathologies such as cancer [8]. 

Several scientific and clinical studies have reported a direct link between impaired glucose tolerance and breast [38] or lung [39] cancer initiation, proliferation and invasiveness due to high circulating glucose levels. The multifactorial origins of diabetes encouraged us to evaluate compounds **3a**–**p** for potential antiproliferative properties against the MCF-7 and the A549 cancer cell lines. Since diabetes requires stable long-term treatment and therefore drugs with significantly reduced or no toxicity, these compounds were also screened for cytotoxicity against the normal HEK293-T cell line. The compounds were evaluated for cytotoxicity in the concentration range comprising 1, 5, 10, 25 and 50 µM, using doxorubicin and gefitinib as reference standards for the assays. Doxorubicin is a broad-spectrum anticancer drug widely used in leukemia, lymphomas and solid tumors such as liver cancer, breast cancer, ovarian cancer, etc. [40]. Gefitinib, on the other hand, has previously been found to induce autophagy in lung cancer A549 cells and to enhance apoptosis and suppress cancer cell proliferation [41]. The IC_50_ values (lethal concentration at which 50% of the cancer cells are killed) of compounds in the **3** series averaged from three independent experiments against doxorubicin and gefitinib are represented in Table 2. The dose–response curve used to calculate IC_50_ values of the test compounds and the percentage cell viability against the MCF-7, A549 and HEK293-T cell lines are listed in Figure S2 and Tables S1–S3 in the Supplementary Material. The test compounds exhibited moderate cytotoxicity against the MCF-7 and A549 cell lines compared to doxorubicin (IC_50_ = 0.25 ± 0.05 μM and 0.36 ± 0.07 μM, respectively) and gefitinib (IC_50_ = 0.19 ± 0.04 μM and 0.25 ± 0.03 μM, respectively), with IC_50_ values in the ranges 10.38 ± 0.08–25.48 ± 0.08 μM and 11.39 ± 0.12–20.00 ± 0.05 μM, respectively. These compounds are, however, less cytotoxic to the HEK293-T cell line compared to doxorubicin (IC_50_ = 0.87 ± 0.04 μM) and gefitinib (IC_50_ = 0.40 ± 0.02 μM), and their IC_50_ values are in the range 20.12 ± 0.06–59.02 ± 0.02 μM. In our view, most of the compounds will probably help to ameliorate complications associated with T2DM with minimal or no cytotoxic effects on normal cells. However, further cytotoxicity tests against different cell lines need to be conducted to confirm the cytotoxicity and/or safety of these compounds.

Considering the effects of oxidative stress on the induction of diabetic complications, these compounds were also evaluated for antioxidant potential in vitro. Several reports recommend the use of multiple methods to evaluate the antioxidant activity of compounds. The initial approach consisted of the evaluation of the ability of these *N*-oxides to scavenge the DPPH and NO radicals. The DPPH and NO radical scavenging activities of compounds **3a**–**p** were evaluated against ascorbic acid (vitamin C) as a reference standard (IC_50_ = 5.02 ± 0.009 µM and 5.39 ± 0.005 µM, respectively) in a concentration range comprising 1, 5, 10, 25 and 50 µM (Table 3). Ascorbic acid has been shown to be an effective scavenger against oxygen and nitrogen oxide species, such as superoxide radical ions, hydrogen peroxide, hydroxyl radical and singlet oxygen. Compound **3a,** with potential dual inhibition of α-glucosidase and α-amylase, also exhibited increased DPPH and moderate NO radical scavenging activities compared to ascorbic acid, with IC_50_ values of 4.74 ± 0.027 µM and 27.34 ± 0.04 µM, respectively. The potential selective α-glucosidase inhibitor **3b**, on the other hand, exhibited increased DPPH and moderate NO radical scavenging activity compared to ascorbic acid, with IC_50_ values of 1.07 ± 0.048 µM and 15.10 ± 0.029 µM, respectively. Significant NO radical scavenging activity (IC_50_ = 7.45 ± 0.002 µM), but reduced DPPH scavenging activity (IC_50_ = 33.52 ± 0.025 µM) were observed for the 2-(4-chlorophenyl)-substituted derivative **3c,** which exhibited strong activity against α-glucosidase (IC_50_ = 0.92 ± 0.012 µM) within the series **3a**–**d**. Among the 6-iodo-substituted derivatives **3e**–**h**, only the 2-phenyl- **3e** and the 2-(4-methoxyphenyl)-substituted derivative **3h** exhibited strong DPPH scavenging activity compared to ascorbic acid, with IC_50_ values of 1.10 ± 0.031 µM and 0.21 ± 0.057 µM, respectively. Compound **3h** also exhibited significant NO scavenging activity (IC_50_ = 10.05 ± 0.003 µM), whereas **3e** was the least active against this radical (IC_50_ = 52.38 ± 0.003 µM) within the category **3e**–**h**. The 2-(4-fluorophenyl)- **3f** and 2-(4-chlorophenyl)-substituted derivative **3g** exhibited significant (IC_50_ = 6.74 ± 0.002 µM) and increased (IC_50_ = 4.25 ± 0.005 µM) NO scavenging activities, respectively. The potential dual α-glucosidase and α-amylase inhibitor **3i** exhibited strong DPPH scavenging activity (IC_50_ = 1.04 ± 0.077 µM) but weak activity against the NO radical (IC_50_ = 61.26 ± 0.005 µM). Only the 2-(4-fluorophenyl)-substituted derivative **3j** showed moderate activity against both the carbohydrate-hydrolyzing enzymes tested and exhibited increased NO scavenging activity (IC_50_ = 5.17 ± 0.007 µM), but the least activity against DPPH (IC_50_ = 61.01 ± 0.053 µM) within the series **3i**–**l**. The presence of the 2-(4-chlorophenyl) group in **3k** resulted in increased DPPH scavenging activity (IC_50_ = 4.08 ± 0.038 µM) and moderate NO scavenging effect (IC_50_ = 15.00 ± 0.005 µM). The 2-(4-fluorophenyl)-substituted derivative **3n** with potential selective inhibitory activity against α-amylase exhibited significant DPPH (IC_50_ = 6.84 ± 0.009 µM) and strong NO scavenging activity (IC_50_ = 0.88 ± 0.004 µM) within the 6-iodo-8-bromo sub-series **3m**–**p**. The presence of a strongly π-electron-delocalizing 4-methoxyphenyl group in **3p** resulted in higher NO radical scavenging activity with an IC_50_ value of 5.46 ± 0.004 µM, which is comparable to that of the reference standard. The most active derivatives against NO radicals, namely, **3c**, **3f**, **3g**, **3j**, **3n** and **3p**, are characterized by the presence of a moderate (F or Cl) or strong (-OCH_3_) π-electron donating group at the *para* position of the 2-phenyl ring. Increased free-radical scavenging activity for these compounds may be due to the interaction of the NO radicals with their additional resonance structures. The DPPH assay has drawbacks, such as partial ionization of the tested compounds and pH dependence [42]. To overcome the disadvantage of a single approach towards NO detection [39], a BioAssay Systems’ EnzyChrom^TM^ Nitric Oxide Synthase Inhibitor Screening Kit (Catalogue No. EINO-100; EnzyChrom) was also used to evaluate derivatives **3c**, **3f**, **3g**, **3j**, **3n** and **3p** for the potential to inhibit NO generated by nitric oxide synthase (NOS) activity. Nitric oxide synthases (NOSs) are a family of enzymes that catalyze the production of NO from L-arginine [43]. This enzyme-based assay is a variant of the Griess assay above and involves two steps. The first of these is a reaction whereby NO is produced from NOS and rapidly oxidized to nitrite (NO_2_^−^) and nitrate (NO_3_^−^). The second (detection) step involves the measurement of NO produced enzymatically from NOS, following the reduction of nitrate to nitrite using the Griess method. Determination of the IC_50_ values for this in vitro NO inhibitory assay was realized in the presence of five different concentrations (1, 5, 10, 25 and 50 μM) of **3a**, **3c**, **3f**, **3i**, **3l**, **3n** or **3p** against ascorbic acid as a reference standard. The results of this assay also confirmed that these compounds have the potential to inhibit NO generated by NOS activity with IC_50_ values in the range 1.10 ± 0.003–29.08 ± 0.004 μM (Table 3). Although ascorbic acid is known to directly scavenge superoxides, preclinical studies have shown that high doses are required for it to inhibit activity of inducible NOS [44]. The observed increased activity of ascorbic acid and moderate to increased activity of compounds **3a**, **3c**, **3f**, **3i**, **3l**, **3n** and **3p** under the current high-dilution conditions was probably due to the direct scavenging of NO generated from NOS activity.

**Figure 3:**
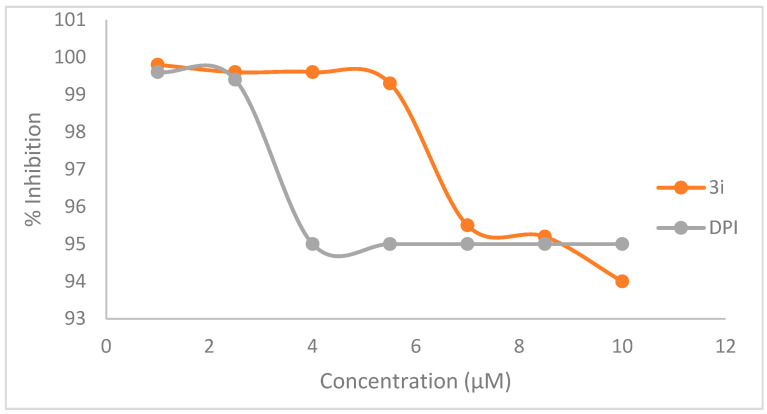
Kinetics of NOS incubated with various concentrations of **3i** and DPI.

In vitro enzymatic generation of reactive species in ROS/RNS (reactive nitrogen species) scavenging assays is complicated by the difficulty of determining whether the enzyme or reactive species is actually inhibited during the reaction [45,46]. As a result, the BioAssay Systems’ EnzyChrom^TM^ Nitric Oxide Synthase Inhibitor Screening assay kit was also used to evaluate if the *N*-oxides also inhibited NOS activity in the first step, during which this enzyme produced NO. Since this reaction is fast, to ensure identical incubation time, only **3i** was subjected to the enzyme-catalyzed kinetic reaction at seven different concentrations (1, 2.5, 4.0, 5.5, 7.0, 8.5 and 10.0 μM) against a specific NOS inhibitor, diphenyleneiodonium chloride (DPI), as a reference standard. The dose–response curves for the two compounds (Figure 3) revealed DPI as the more potent NOS inhibitor at low concentrations (1–4 μM), with an IC_50_ value of 3.00 ± 0.007 μM. NOS activity of either a semipurified enzyme preparation or activated macrophage cytosol was previously irreversibly inhibited after a 30 min. incubation period with 10 μM DPI at 37 °C [47]. Compound **3i** (IC_50_ = 6.75 ± 0.002 μM), in our view, will probably impact the enzyme activity at relatively higher concentrations and still produce an effect on the second reaction.

The metabolic abnormalities of diabetes cause mitochondrial superoxide overproduction, the main mediator of diabetes tissue damage; insulin resistance; β-cell dysfunction; and impaired glucose tolerance [48]. Highly reactive superoxide radicals are routinely produced by cells and detoxified by superoxide dismutase (SOD), which catalyzes the conversion of O_2_^•−^ into H_2_O_2_ and O_2_, thereby regulating ROS generation [49]. This enzyme competes with NO for superoxide anions and inactivates NO to form peroxynitrite. This reaction is accompanied by alternate oxidation–reduction of metal ions, such as Cu^2+^, Zn^2+^, Fe^2+^ and Mn^2+^, which are vital to enzymatic activity and are present in the active site of SOD [50]. SOD constitutes a very important antioxidant defense against oxidative stress in the body, and its inhibition leads to free radical-mediated damage to mitochondrial membranes and the release of cytochrome c. Compounds **3a**, **3c**, **3f**, **3i**, **3l**, **3n** and **3p** were evaluated via enzymatic assay in vitro for potential to inhibit or activate the antioxidant activity of SOD using DPI as a reference standard for the assay. DPI is a known specific inhibitor of SOD and affects mitochondrial function. Figure S3 in the Supplementary Materials contains graphs of % inhibition of SOD used to calculate the IC_50_ values of these compounds. Compounds **3a, 3f** and **3i** exhibited reduced SOD inhibitory activity compared to DPI (IC_50_ = 0.71 ± 0.03 µM), with IC_50_ values of 15.02 ± 010, 11.59 ± 0.08 and 15.00 ± 0.14 µM, respectively. Though significantly less inhibiting compared to DPI, derivatives **3c** (4.60 ± 0.14 µM), **3l** (5.60 ± 0.17 µM), **3n** (6.20 ± 0.12 µM) and **3p** (8.30 ± 0.04 µM) are moderately inhibiting against SOD activity. Compounds **3a**, **3f** and **3i**, with potential dual inhibitory effect against α-glucosidase and α-amylase, strong free radical scavenging properties and reduced toxicity against the normal cell line as well as reduced inhibitory activity against SOD, will normalize postprandial hyperglycemia and prevent complications of T2DM. The observed reduced toxicity of the test compounds against the normal cell line is probably due to the inhibition of the reactive oxygen species which are associated with toxicity.

**Scheme 2:**
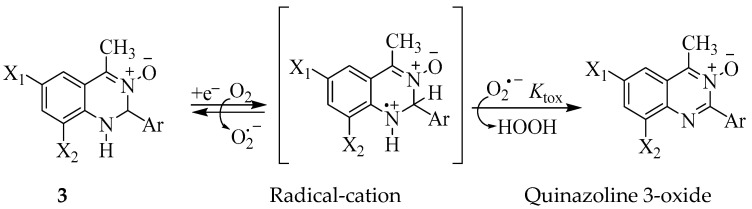
Proposed mechanism for the free radical scavenging reaction of **3** in vitro.

The observed free radical scavenging properties of the test compounds prompted us to propose a mechanism outlined in Scheme 2. It is envisaged that the superoxide radical (O_2_^•−^) formed under aerobic (high oxygen concentration) or hypoxic (low [O_2_]) conditions in the cells will convert the electron-rich amine nitrogen of **3** into a radical-cation center. A radical-cation intermediate was previously implicated in the mechanism of the visible light photo-redox catalysis of the 2-aryl-1,2-dihydroquinazoline 3-oxides in the presence of 0.5 mol % tris(bipyridine)ruthenium(II) chloride (Ru(bpy)_3_Cl_2_) as a photocatalyst in acetonitrile under aerobic conditions [31]. On the other hand, the analogous heteroaromatic *N*-oxides such as 3-amino-1,2,4-benzotriazine 1,4-dioxide (tirapazamine), for example, are envisaged to react with superoxide radical under aerobic conditions in vivo to form radical-anion intermediates [9]. Under aerobic conditions, the kinetically favored pathway will involve the reduction of the resonance-stabilized radical-cation intermediate back to the parent compound followed by back-oxidation with the corresponding generation of O_2_^•−^. On the other hand, the apparent rate constant of the back-oxidation reaction (defined as *k*O_2_[O_2_]) will decrease under hypoxic conditions, in turn prolonging the lifetime of the drug radical-cation [51]. The superoxide radical scavenging effect of the test compounds could culminate in the prevention of the hydroxyl (^•^OH) radical formation. An alternative slower process (*k*_tox_) [51] which may involve proton and hydrogen transfers from the radical-cation to superoxide anion radical would furnish quinazoline 3-oxide and hydrogen peroxide as a by-product. This will probably stop the oxidation chain reaction and prevent oxidative stress-related damage.

Antioxidant activity may easily be demonstrated in vitro; however, it is much more difficult to prove that the reaction is also relevant in vivo. Nevertheless, the test compounds that exhibited inhibitory effects against the carbohydrate hydrolyzing enzymes and potent antioxidant activities may be very good candidates for controlling oxidative stress in diabetic patients.

### 3.3. Computational Studies 

Hydrogen bonds, halogen bonds, aromatic–aromatic (π···π, C–H···π stacking, etc.) and electrostatic (π-cation/anion, charged) interactions, as well as weak van der Waals or dipole–dipole interactions, are of interest to medicinal chemists [52]. These non-covalent interactions help to tailor the solubility, stability, dissolution rate and bioavailability of drug molecules and to extrapolate SAR [53]. Non-covalent and hydrophobic interactions, including alkyl, π-π and π-alkyl interactions between inhibitors and α-glucosidase [54] or α-amylase [55] have been found to enhance the inhibitory activities of these enzymes. It is envisaged that the presence of potential hydrogen bond donating NH and accepting N-O as well as halogen atoms on the scaffold of these compounds further stabilized the interactions of drug molecules with the receptors. The most active derivative/s from each series against α-glucosidase and/or α-amylase were subjected to molecular docking to reveal their plausible binding modes in the active sites of these enzymes to correlate the results of in silico and in vitro studies to guide further ligand design. 

#### 3.3.1. Molecular Docking **3a**, **3c**, **3f**, **3i**, **3l** and **3p** into α-Glucosidase and α-Amylase

**Figure 4:**
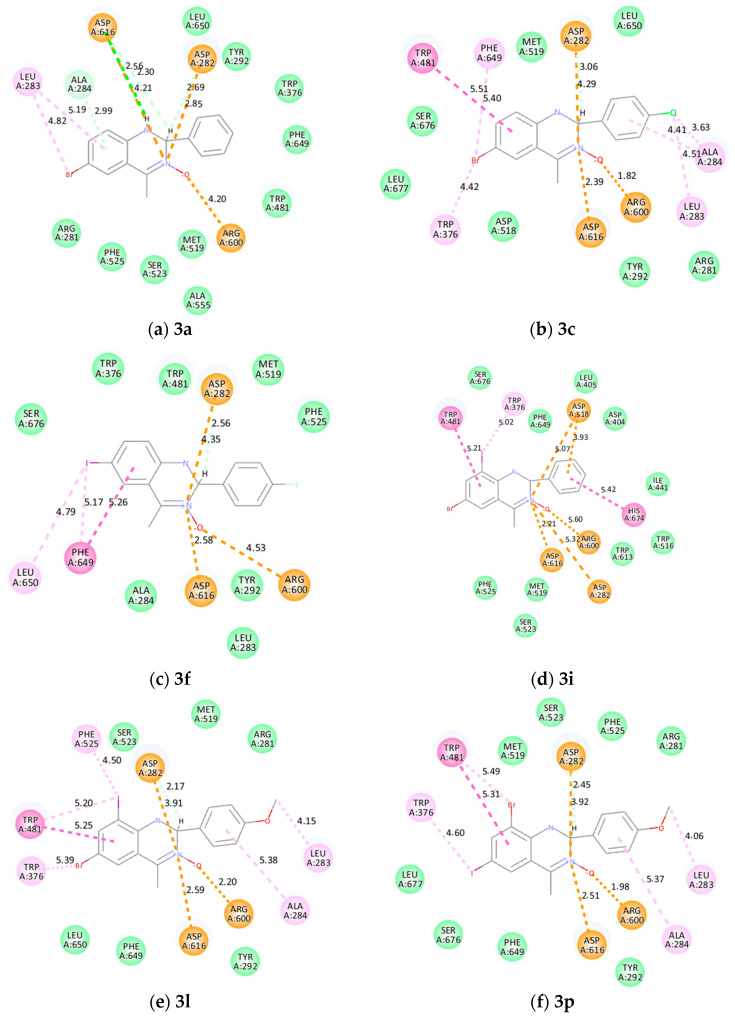
Molecular docking of compounds (**a**) **3a**, (**b**) **3c**, (**c**) **3f**, (**d**) **3i**, (**e**) **3l** and (**f**) **3p** into α-glucosidase. Interaction types are marked with dashed lines and are color-coded as follows: bright green: conventional hydrogen bond; lighter green: van der Waals interaction; very light green: carbon–hydrogen bond; orange: a salt bridge or attractive charge or pi-anion; bright pink: π-π T-shaped; light pink: alkyl or π-alkyl.

The calculated binding energies (kcal/mol) for these compounds are listed in Table S5 of the Supplementary Material. The binding energy values obtained via molecular docking do not always correlate well with the biological response observed in the in vitro tests. A limitation of molecular docking is the lack of confidence in scoring functions’ ability to provide correct binding energies, because some intermolecular interaction terms, such as the solvation effect and entropy change, are difficult to predict precisely [56]. Nevertheless, molecular docking is an important in silico technique used to predict the interaction between the receptor and the ligand. The examination of docked complexes was carried out with Discovery Studio 2021 molecular visualization software, which represented the 2D interactions between ligand atoms and specific amino acids in the active site of the receptor. Special attention was given to the ones (**3a**, **3c**, **3f**, **3i**, **3l** and **3p**) exhibiting a higher inhibitory effect against α-glucosidase and/or α-amylase in their groups. Although their inhibitory activities were evaluated using α-glucosidase from the *Saccharomyces cerevisiae*, the compounds were docked into the human lysosomal acid α-glucosidase (PDB CODE: 5NN8). Acarbose (BE = −35.1447) docked into the active site like the native ligand, to engage in several hydrogen bonding interactions with Asp282, Asp616 and Arg600 as well as attractive charge interaction with Asp616 (Figure S5a). The active site residues such as Asp518 and Asp616 acted as a catalytic nucleophile and acid/base donors, respectively [57]. Figure 4a shows **3a** fitting very well in the acarbose binding cavity, and its fused benzo ring and bromine atom are involved in π-alkyl (CH–π) and alkyl interactions with Leu283, respectively. The fused benzo ring is also involved in a π-donor hydrogen bonding interaction with Ala284. The nitrogen atom of the N-O moiety is involved in charge interactions with Asp282 and Asp616. Both residues are also involved in weak carbon–hydrogen bonding interaction with the hydrogen atom of the methine group. Conventional hydrogen bonding interaction is predicted between Asp616 and NH. The oxygen atom of the N-O moiety, on the other hand, forms a salt bridge with Arg600. Hydrophobic and hydrogen bonding and charged interactions stabilize the interactions between this compound and α-glucosidase to enhance its inhibitory activity against this enzyme. The fused benzo ring of **3c** (Figure 4b) is involved in a π-π T-shaped interaction with Trp481. The bromine atom is involved in π-alkyl interaction with Phe649 and Trp376, respectively. The nitrogen atom of the N-O moiety is also involved in charge interactions with Asp282 and Asp616, while the oxygen atom interacts with Arg600 to form a salt bridge. The ring of the 4-chlorophenyl group is involved in a π-alkyl interaction with Ala284 while this residue and Leu283 form alkyl interactions with the chlorine atom. A weak carbon–hydrogen bond exists between the hydrogen atom of the methine group and Asp282. The presence of bromine and chlorine atoms resulted in an increased number of hydrophobic interactions with several residues in the active site of this enzyme. These interactions probably account for the increased inhibitory effect of this compound against α-glucosidase compared to **3a**. There is a π-π T-shaped interaction between the fused benzo ring of **3f** (Figure 4c) and Phe649, and the iodine atom is involved in alkyl and π-alkyl interactions with Leu650 and Phe649, respectively. No hydrogen bonding interaction is predicted between NH and any of the residues in the active pocket, which may account for the reduced inhibitory activity of this compound compared to **3a**. The fused benzo and phenyl rings of **3i** (Figure 4d) are involved in π-π T-shaped interactions with Trp481 and His674, respectively. The phenyl ring is also involved in a π-anion interaction with Asp518. A π-alkyl interaction exists between the iodine atom and Trp376. The nitrogen atom of N-O moiety is predicted to be involved in attractive charge interactions with the residues Asp282 and Asp616 and also a π-anion interaction with Asp518. The oxygen atom forms a salt bridge with Arg600. Hydrophobic and increased electrostatic interactions appear to stabilize the interactions of this compound with α-glucosidase to enhance its inhibitory effect. The fused benzo ring of **3l** (Figure 4e) is involved in a π-π T-shaped interaction with Trp481. The bromine atom is involved in a π-alkyl interaction with Trp376, and similar interactions exist between iodine atom and the residues Trp481 and Phe525. The residues Asp282 and Asp616 are involved in charge interaction with the nitrogen atom of the N-O moiety. The oxygen atom of this moiety forms a salt bridge with Arg600. A π-alkyl interaction is implicated between the iodine atom of **3p** (Figure 4f) and the residues Trp376. The fused benzo ring itself is involved in a π-π T-shaped stacking interaction with Trp481. Attractive charge interactions involve nitrogen of the N-O moiety and the residues Asp282 and Asp616, while the oxygen atom forms a salt bridge with Arg600. The methine hydrogen atom of this ligand is involved in a weak carbon–hydrogen bonding interaction with Asp282. The ring of the 4-methoxyphenyl group is involved in a π-alkyl interaction with Ala284, while Leu283 is involved in alkyl interaction with carbon atom of the methoxy group. Increased hydrophobic (alkyl, π-alkyl and π-π T-shaped) interactions due to the presence of halogen atoms on the fused benzo ring, and the 4-methoxyphenyl group helped to embed **3l** and **3p** in the active site of α-glucosidase and increased their activity against this enzyme. These findings are in good agreement with previous studies [56] which showed that non-covalent and hydrophobic interactions influence the inhibitory effect of ligands against α-glucosidase activity. The N-O moiety facilitated electrostatic interactions of the test compounds with the protein residues in the active site of α-glucosidase, such as Asp282, Arg600 and Asp616. Although no halogen bonding interactions were predicted, the presence of bromine and/or iodine on the fused benzo ring helped to increase the number of hydrophobic interactions with protein residues in the active site of α-glucosidase which were involved in α-glucosidase-acarbose complex formation. Acarbose is a known competitive inhibitor of α-glucosidase, which binds to the active site of the protein, and thus these heterocycle *N*-oxides are potential competitive inhibitors of this enzyme. 

**Figure 5:**
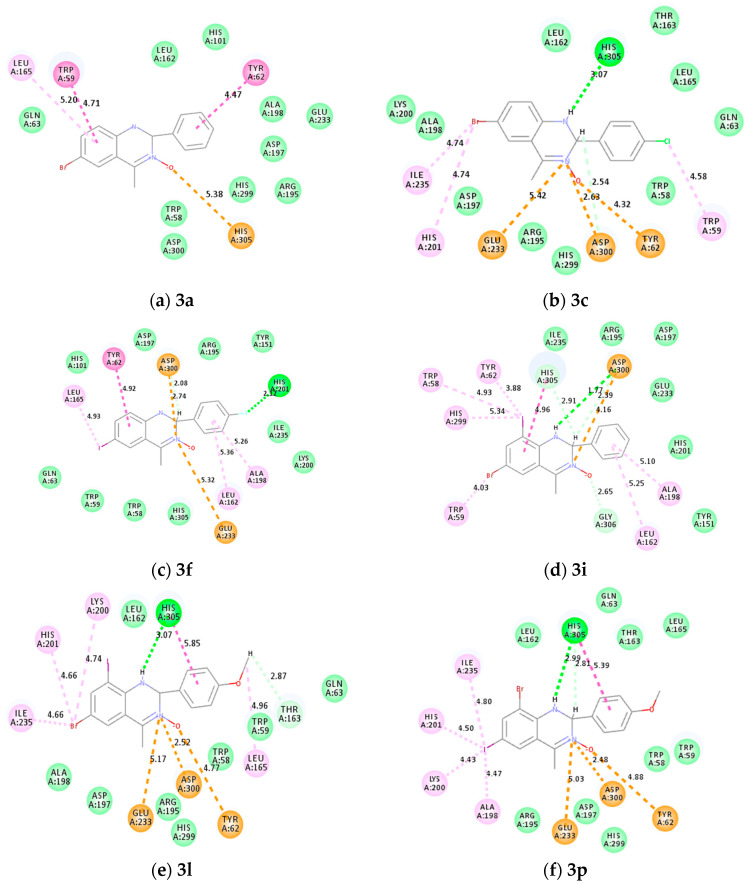
Molecular docking of compounds (**a**) **3a**, (**b**) **3c**, (**c**) **3f**, (**d**) **3i**, (**e**) **3l** and (**f**) **3p** into α-amylase. Interaction types are marked with dashed lines and are color-coded as follows: bright green: conventional hydrogen bond; lighter green: van der Waals interaction; very light green: carbon–hydrogen bond; orange: a salt bridge or attractive charge or pi-anion; bright pink: π-π T-shaped; light pink: alkyl or π-alkyl.

The docking pose of acarbose (BE = −25.2018 kcal/mol) into human pancreatic α-amylase (PDB code 3BAJ) revealed the presence of hydrogen bonding interactions with the residues Thr151, Glu240, His201, His299, His305, Asp197 and Asp300 in the active site of α-amylase (Figure S4). The docking poses of **3a**, **3c**, **3f**, **3i**, **3l** and **3p** into this active site of this enzyme are represented in Figure 5. The fused benzo ring and the phenyl ring of **3a** (Figure 5a) are involved in π-π stacked interactions with Trp59 and Tyr62, respectively. Leu165 forms a π-alkyl interaction with the fused benzo ring. The oxygen atom of the N-O moiety is involved in an attractive charge and π-anion interactions with His305. Despite fitting well within the binding pocket of this enzyme, this compound did not interact with Asp197, Glu233 or Asp300 of the catalytic triad, which were involved in α-amylase-acarbose complex formation. Asp197 acted as a nucleophile, while Glu233 acted in acid–base catalysis during the hydrolysis reaction, and Asp300 acted as a key residue in optimizing the orientation of the substrate [58]. Attractive charge interactions existed between nitrogen and oxygen atoms of the N-O moiety of **3c** (Figure 5b) with Glu233 and Asp300 of the catalytic triad. The oxygen atom of this moiety is also involved in a π-anion interaction with Tyr62. The NH and the hydrogen atom of the methine group engaged in hydrogen bonding and weak bonding interactions with His305 and Asp300, respectively. Trp59 is involved in alkyl interaction with the chlorine atom of this compound. Hydrophobic and electrostatic interactions seemed to enhance the inhibitory activity of this compound against α-amylase. The iodine and fluorine atoms of **3f** (Figure 5c) were involved in alkyl and hydrogen bonding interactions with Leu165 and His205, respectively. A π-π T-shaped interaction was predicted between the fused benzo ring and Tyr62. The nitrogen atom of the N-O moiety was involved in charge interactions with Glu233 and Asp300 of the catalytic triad. A weak carbon–hydrogen bond is predicted between the hydrogen atom of the methine group with Asp300. The ring of the 4-fluorophenyl group was involved in π-alkyl interactions with the residues Leu162 and Ala198. Increased noncovalent, hydrophobic and electrostatic interactions of this compound with the protein residues in the catalytic site of this enzyme probably accounted for its strong inhibitory effect against α-amylase activity. The bromine atom of **3i** (Figure 5d) was involved in a π-alkyl interaction with Trp59. The presence of iodine atom resulted in increased π-alkyl interactions involving the residues His299, Trp58 and Tyr62. The fused benzo ring formed a π-π T-shaped interaction with His305, whereas the phenyl ring was involved in π-alkyl interactions with Ala198 and Leu162. The hydrogen atom of the amine group was involved in a conventional hydrogen bonding interaction with Asp300, which might explain the higher inhibitory activity observed for this derivative. Weak carbon–hydrogen bonding interactions were predicted between the methine proton and the residues His305 and Asp300. The latter residue of the catalytic triad was also involved in a charge interaction with nitrogen atom of the N-O moiety. This compound also exhibited increased noncovalent, hydrophobic and electrostatic interactions with the protein residues in the catalytic site consistent with its strong inhibitory activity against α-amylase. The bromine atom of **3l** (Figure 5e) was involved in π-alkyl interactions with Ile235, His201 and Lys200. The nitrogen atom of the N-O moiety was involved in charge interactions with Glu233 and Asp300 and the oxygen atom in π-anion interaction with Tyr62. A hydrogen bonding interaction existed between NH and the residue His305, which was also involved in π-π stacked interaction with the ring of the 4-methoxyphenyl group. The carbon and hydrogen atoms of the methoxy group were involved in an alkyl and a weak carbon–hydrogen bonding interaction with Leu165 and Thr163, respectively. The iodine atom of this compound was not involved in any interaction with the residues in the catalytic site, which may account for its reduced activity against this enzyme. The interactions between 6-iodo-8-bromo substituted **3p** (Figure 5f) and α-amylase were nearly the same as those of isomeric 6-bromo-8-iodo substituted **3l**. The main difference was that the methoxy group of **3l** formed extra interactions, which probably made this ligand more active against α-amylase compared to **3p**. Despite increased interactions of **3c**, **3l** and **3p** with amino acids in the active site of α-amylase, these compounds were less inhibiting against this enzyme compared to the analogous **3a**, **3f** and **3i** with relatively fewer interactions. It has been established that the non-covalent and hydrophobic (alkyl, π-π and π-alkyl) interactions between inhibitors and α-amylase enhance the inhibitory activity of this enzyme [57]. The protein−ligand binding interactions of amino acids such as Phe, Tyr and Trp frequently involve π-π, π-cation and CH-π interactions contributing to protein stability, protein–ligand interactions, catalysis and self-assembly [59]. Molecular docking revealed that the fused benzo ring of **3a** and **3f** were involved in π-π T-shaped interaction with Tyr62, whereas this residue in the case of **3i** was involved in π-alkyl interaction with the iodine atom. The docking poses of **3c**, **3l** and **3p**, on the other hand, revealed Tyr62 to be involved π-anion interaction with oxygen atom of the N-O moiety, which probably accounts for the reduced activity against α-amylase.

#### 3.3.2. Pharmacokinetics Properties Prediction of **3a**–**p**

To better understand their pharmacokinetics and drug-likeness properties, the SwissADME tool was used to predict the absorption, distribution, metabolism and excretion (ADME) properties of the test compounds (Table 4). All compounds scored within the desired ranges for five (lipophilicity, molecular weight or size, polarity, solubility and flexibility) of the six bioavailability scores. Compounds **3a**, **3d** and **3h** did not violate any of the Lipinski rules of five (Ro5), while the other derivatives violated one, with the log of the partition coefficient (mLogP) being greater than 4.15 (Table 4). The test compounds scored out of the optimal range for saturation, which is the fraction of carbons in the sp^3^ hybridization not being less than 0.25. Flexibility is measured by the number of rotatable bonds, and these should not exceed 9. Derivatives **3c**, **3g**, **3k** and **3o** substituted at the 2-position with a 4-chlorophenyl group are predicted to exhibit increased lipophilicity, consistent with the chlorine effect [37].

## 4. Conclusions

The results of this study highlight the bench-stable 6-bromo/iodo and their mixed 6,8-dihalogeno substituted 2-aryl-4-methyl-1,2-dihydroquinazoline 3-oxides **3** as potential inhibitors of carbohydrate-hydrolyzing enzymes with significant to strong antioxidant activities. Although the compounds exhibited moderate cytotoxicity against the MCF-7 and/or A549 cell line, they were less effective against the viability of the HEK-293-T cells. The strong activity of **3c**, **3l** and **3p** against α-glucosidase and the potential dual inhibitory effect of **3a**, **3f** and **3i** against these enzymes will probably help to ameliorate complications associated with T2DM with minimal or no cytotoxic effects on normal cells. Their significant to strong free radical scavenging potential will enable them to delay, inhibit or prevent oxidative damage by scavenging free radicals. Molecular docking suggests that hydrophobic and electrostatic interactions as well as hydrophilic interactions are important for the inhibitory activity of these compounds against carbohydrate-hydrolyzing enzymes. The presence of bromine and/or iodine on the fused benzo ring helped to increase the number of hydrophobic interactions, resulting in a strong inhibitory effect against α-glucosidase and/or α-amylase activities. The present study suggests enzyme inhibition and antioxidant activity as potential pathways of the 1,2-dihydroquinazoline 3-oxides in the treatment of diabetes. However, the in vitro chemical assays bear no similarity to biological systems and do not take into account the compound’s bioavailability. Since this is a preliminary study, further cellular-based in vitro and in vivo studies including bioavailability and cell permeability are required to help clarify the mechanism of action of these compounds in the body. This will also help to establish their safety profile as potential multi-target agents against the pathogenesis and progression of T2DM.

## Figures and Tables

**Table 1:** Substitution pattern and designation of groups X_1_, X_2_ and Ar as well as % yield of **3a**–**p**.

3a–p	X_1_	X_2_	Ar	% Yield
**3a**	Br	H	-C_6_H_5_	62
**3b**	Br	H	-C_6_H_4_(4-F)	50
**3c**	Br	H	-C_6_H_4_(4-Cl)	84
**3d**	Br	H	-C_6_H_4_(4-OCH_3_)	90
**3e**	I	H	-C_6_H_5_	68
**3f**	I	H	-C_6_H_4_(4-F)	72
**3g**	I	H	-C_6_H_4_(4-Cl)	88
**3h**	I	H	-C_6_H_4_(4-OCH_3_)	86
**3i**	Br	I	-C_6_H_5_	74
**3j**	Br	I	-C_6_H_4_(4-F)	60
**3k**	Br	I	-C_6_H_4_(4-Cl)	80
**3l**	Br	I	-C_6_H_4_(4-OCH_3_)	84
**3m**	I	Br	-C_6_H_5_	76
**3n**	I	Br	-C_6_H_4_(4-F)	66
**3o**	I	Br	-C_6_H_4_(4-Cl)	98
**3p**	I	Br	-C_6_H_4_(4-OCH_3_)	94

**Table 2:** IC_50_ values of **3a**–**p** against α-glucosidase, α-amylase, MCF-7, A549 and HEK293-T.

	IC_50_ (µM ± SD)				
3a–p	α-Glucosidase	α-Amylase	MCF-7	A549	Hek293-T
**3a**	1.08 ± 0.02	5.33 ± 0.01	10.83 ± 0.09	15.84 ± 0.07	40.18 ± 0.02
**3b**	7.47 ± 0.05	36.35 ± 0.01	12.80 ± 0.07	19.31 ± 0.08	41.72 ± 0.04
**3c**	0.92 ± 0.01	30.48 ± 0.02	25.48 ± 0.08	15.26 ± 0.09	27.54 ± 0.05
**3d**	68.2 ± 0.01	54.08 ± 0.03	13.68 ± 0.09	13.45 ± 0.03	37.53 ± 0.18
**3e**	6.04 ± 0.01	49.50 ± 0.06	15.83 ± 0.14	11.39 ± 0.12	20.12 ± 0.06
**3f**	9.27 ± 0.02	0.64 ± 0.01	19.01 ± 0.04	13.71 ± 0.11	22.32 ± 0.08
**3g**	15.08 ± 0.08	53.42 ± 0.01	10.38 ± 0.08	18.63 ± 0.11	21.70 ± 0.13
**3h**	27.18 ± 0.02	55.72 ± 0.02	22.62 ± 0.12	18.71 ± 0.16	24.26 ± 0.04
**3i**	1.01 ± 0.05	1.18 ± 0.06	15.43 ± 0.06	16.85 ± 0.05	34.90 ± 0.13
**3j**	19.69 ± 0.01	17.48 ± 0.03	15.06 ± 0.01	13.70 ± 0.12	32.83 ± 0.06
**3k**	13.47 ± 0.04	4.46 ± 0.02	16.65 ± 0.05	15.37 ± 0.13	21.04 ± 0.05
**3l**	1.04 ± 0.03	54.18 ± 0.01	18.12± 0.10	16.34 ± 0.06	32.39 ± 0.17
**3m**	9.14 ± 0.03	16.73 ± 0.01	15.34 ± 0.07	16.34 ± 0.06	27.97 ± 0.08
**3n**	43.23 ± 0.05	4.71 ± 0.01	14.79 ± 0.10	17.22 ± 0.07	32.42 ± 0.06
**3o**	7.07 ± 0.04	49.18 ± 0.01	16.50 ± 0.01	20.00 ± 0.05	58.98 ± 0.17
**3p**	0.78 ± 0.05	73.66 ± 0.02	13.47 ± 0.06	12.24 ± 0.02	59.02 ± 0.02
Acarbose	4.40 ± 0.04	2.92 ± 0.02	-	-	-
Doxorubicin	-	-	0.25 ± 0.05	0.36 ± 0.07	0.87 ± 0.04
Gefitinib	-	-	0.19 ± 0.04	0.25 ± 0.03	0.40 ± 0.02

**Table 3:** IC_50_ values for the DPPH and NO radical scavenging activities of compounds **3a**–**p**.

		IC_50_ (µM ± SD)	
3a–p	DPPH	NO	NO from NOS
**3a**	4.74 ± 0.027	27.34 ± 0.04	5.24 ± 0.002
**3b**	1.07 ± 0.048	15.10 ± 0.029	-
**3c**	33.52 ± 0.025	7.45 ± 0.002	1.10 ± 0.003
**3d**	9.55 ± 0.006	24.42 ± 0.004	-
**3e**	1.10 ± 0.031	52.38 ± 0.003	-
**3f**	23.66 ± 0.009	6.74 ± 0.002	4.70 ± 0.004
**3g**	26.88 ± 0.043	4.25 ± 0.005	-
**3h**	0.21 ± 0.057	10.05 ± 0.003	-
**3i**	1.04 ± 0.077	61.26 ± 0.005	29.08 ± 0.004
**3j**	61.01 ± 0.053	5.17 ± 0.007	-
**3k**	4.08 ± 0.038	15.00 ± 0.005	-
**3l**	27.70 ± 0.040	67.81 ± 0.004	1.06 ± 0.005
**3m**	8.88 ± 0.011	55.39 ± 0.002	-
**3n**	6.84 ± 0.009	0.88 ± 0.004	3.50 ± 0.01
**3o**	88.45 ± 0.009	23.25 ± 0.002	-
**3p**	60.03 ± 0.014	5.46 ± 0.004	15.51 ± 0.009
Ascorbic acid	5.02 ± 0.009	5.39 ± 0.005	0.999 ± 0.006

**Table 4:** Pharmacokinetics properties predictions of compounds **3a**–**p**.

Property	Compound															
	3a	3b	3c	3d	3e	3f	3g	3h	3i	3j	3k	3l	3m	3n	3o	3p
Lipophilicity (XLogP3)	2.64	2.74	3.27	2.61	2.60	2.70	3.23	2.57	3.29	3.39	3.92	3.26	3.29	3.39	3.92	3.26
Polarity-TPSA (Å)	40.78	40.78	40.78	50.01	40.78	40.78	40.78	50.01	40.78	40.78	40.78	50.01	40.78	40.78	40.78	50.01
Solubility (Log S)	−3.87	−4.02	−4.46	−3.93	−4.14	−4.29	−4.72	−4.19	−5.04	−5.19	−5.63	−5.10	−5.04	−5.19	−5.63	−5.10
GI Absorption	High	High	High	High	High	High	High	High	High	High	High	High	High	High	High	High
Saturation (fraction Csp^3^)	0.13	0.13	0.13	0.19	0.13	0.13	0.13	0.19	0.13	0.13	0.13	0.19	0.13	0.13	0.13	0.19
Molecular weight	317.18	335.17	351.63	347.21	364.18	382.17	398.63	394.21	443.08	461.07	477.52	473.10	443.08	461.07	477.52	473.10
mLogP	4.12	4.51	4.63	3.77	4.24	4.63	4.74	3.89	4.86	5.24	5.36	4.51	4.86	5.24	5.36	4.51
Hydrogen bond acceptor	1	2	1	2	1	2	1	2	1	2	1	2	1	2	1	2
Hydrogen bond donor	1	1	1	1	1	1	1	1	1	1	1	1	1	1	1	1
Rotatable bonds	1	1	1	2	1	1	1	2	1	1	1	2	1	1	1	2
Lipinski’s violation	None	Yes 1	Yes 1	None	Yes 1	Yes 1	Yes 1	None	Yes 1	Yes 1	Yes 1	Yes 1	Yes 1	Yes 1	Yes 1	Yes 1

## Data Availability

The CIF file containing complete information on the studied structure was deposited with the Cambridge Crystallographic Data Center, CCDC 2298438, and is freely available upon request from the following website: www.ccdc.cam.ac.uk (accessed on 25 September 2023) or by contacting the Cambridge Crystallographic Data Center, 12, Union Road, Cambridge CB2 1EZ, UK; fax: +44-1223-336033; email: deposit@ccdc.cam.ac.u.

## Supplementary Materials

The following supporting information can be downloaded at https://www.mdpi.com/article/10.3390/antiox12111971/s1, Figure S1: Analytical data and copies of the ^1^H-NMR and ^13^C-NMR spectra of compounds **2a**–**d** and **3a**–**p**; Table S1: X-ray analysis, crystal data and structure refinement for **3i**; Figure S2: graphs showing dose-dependent effect on cell viability in MCF-7, A549 and HEK293-T cell lines; Table S2: cell viability percentage of compounds **3a**–**p** against MCF-7; Table S3: cell viability percentage of compounds **3a**–**p** against the and A549 cell lines; Table S4: cell viability percentage of compounds **3a**–**p** against the HEK293-T cell line; Figure S3: graphs of % inhibition of SOD used to calculate IC_50_ values; Table S5: calculated binding energies for acarbose and selected compounds **3**; and Figure S4: docking poses of acarbose against α-glucosidase and α-amylase. References [60,61,62,63,64,65,66] are cited in the Supplementary Materials.
