# Supplementary material for: In Vitro α-Glucosidase and α-Amylase Inhibition, Cytotoxicity and Free Radical Scavenging Profiling of the 6-Halogeno and Mixed 6,8-Dihalogenated 2-Aryl-4-methyl-1,2-dihydroquinazoline 3-Oxides
Source: Antioxidants (Basel). 2023 Nov 6;12(11):1971. doi: 10.3390/antiox12111971 (PMC10669220; doi:10.3390/antiox12111971)
Supplement: Supplementary file 1 [file antioxidants-12-01971-s001.zip › antioxidants-2668611-supplementary.pdf]

In Vitro α-Glucosidase and α-Amylase Inhibition, Cytotoxicity and Free Radical Scavenging Profiling of the 6-Halogeno and Mixed 6,8-Dihalogenated 2-Aryl-4-methyl-1,2-dihydroquinazoline 3-oxides

N.M. Magwaza, G.K. More, S. Gildenhuys and M.J. Mphahlele

Fig. S1: Analytical data for compounds **2a–d** and **3a–p**
Table S1: X-ray analysis, crystal data and structure refinement for **3i**
Fig. S2: Graphs showing dose-dependent effect on cell viability in HEK293-T, MCF-7 and A549
Table S2: Cell viability percentage of compounds **3a–p** against MCF-7 cell line
Table S3: Cell viability percentage of compounds **3a–p** against the A549 cell line
Table S4: Cell viability percentage of compounds **3a–p** against the HEK293-T cell line
Fig. S3: Graphs of % **i**nhibition of SOD used to calculate IC_{50} values
Table S5: Binding energies of **3a, 3c, 3f, 3i, 3l** and **3p** docked into α-glucosidase and α-amylase
Fig. S4: Docking poses of acarbose against α-glucosidase and α-amylase

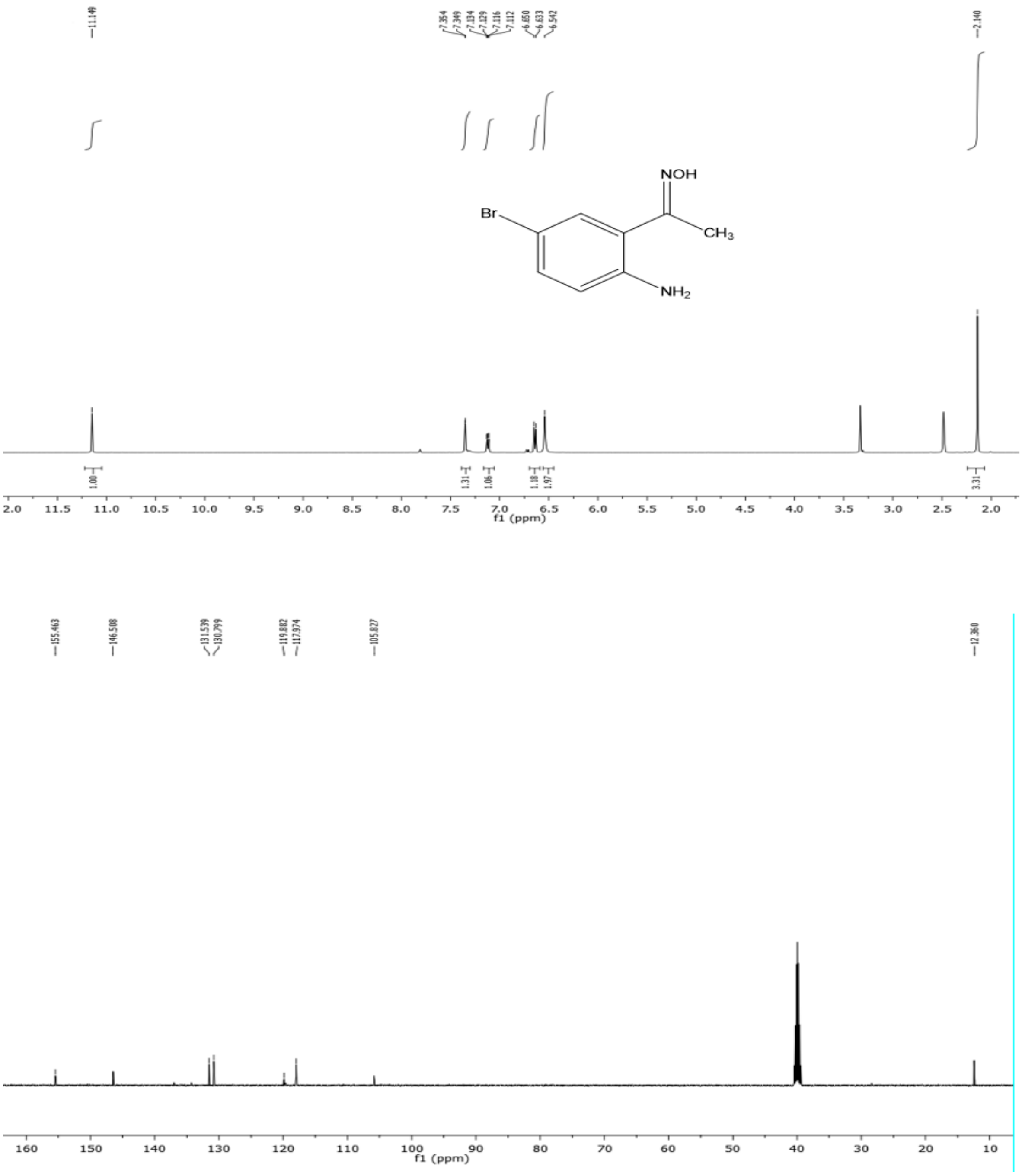

Figure S1.1: ^{1}H- and ^{13}C-NMR spectra of **2a** in DMSO-d_6 at 500 MHz and 125 MHz, respectively

1-(2-Amino-5-bromophenyl)ethan-1-one oxime (**2a**)

White solid (1.85 g, 93%), mp. 159–160 °C (Lit. 161.0–161.5 °C [1]); ν_{max} (ATR): 822, 1002, 1278, 1396, 1488, 1615, 2810, 3248, 3349 cm^{-1}; ^{1}H-NMR (500 MHz, DMSO-d_6): δ 2.30 (s, 3H, -CH3), 6.60 (s, 2H, -NH_2), 7.09 (d, *J* = 6.0 Hz, 1H, H-3), 7.36 (t, *J* = 7.5 Hz, 1H, H-4), 7.42 (d, *J* = 6.0 Hz, 1H, H-6), 7.83 (s, 1H, OH); ^{13}C-NMR (125 MHz, DMSO-d_6): δ 12.5, 108.5, 120.6, 131.1, 132.0, 137.2, 144.6, 157.4; HRMS (ES): MH^{+}, calcd for $C_8H_9N_2OBr$: 228.9898; found 228.9974.

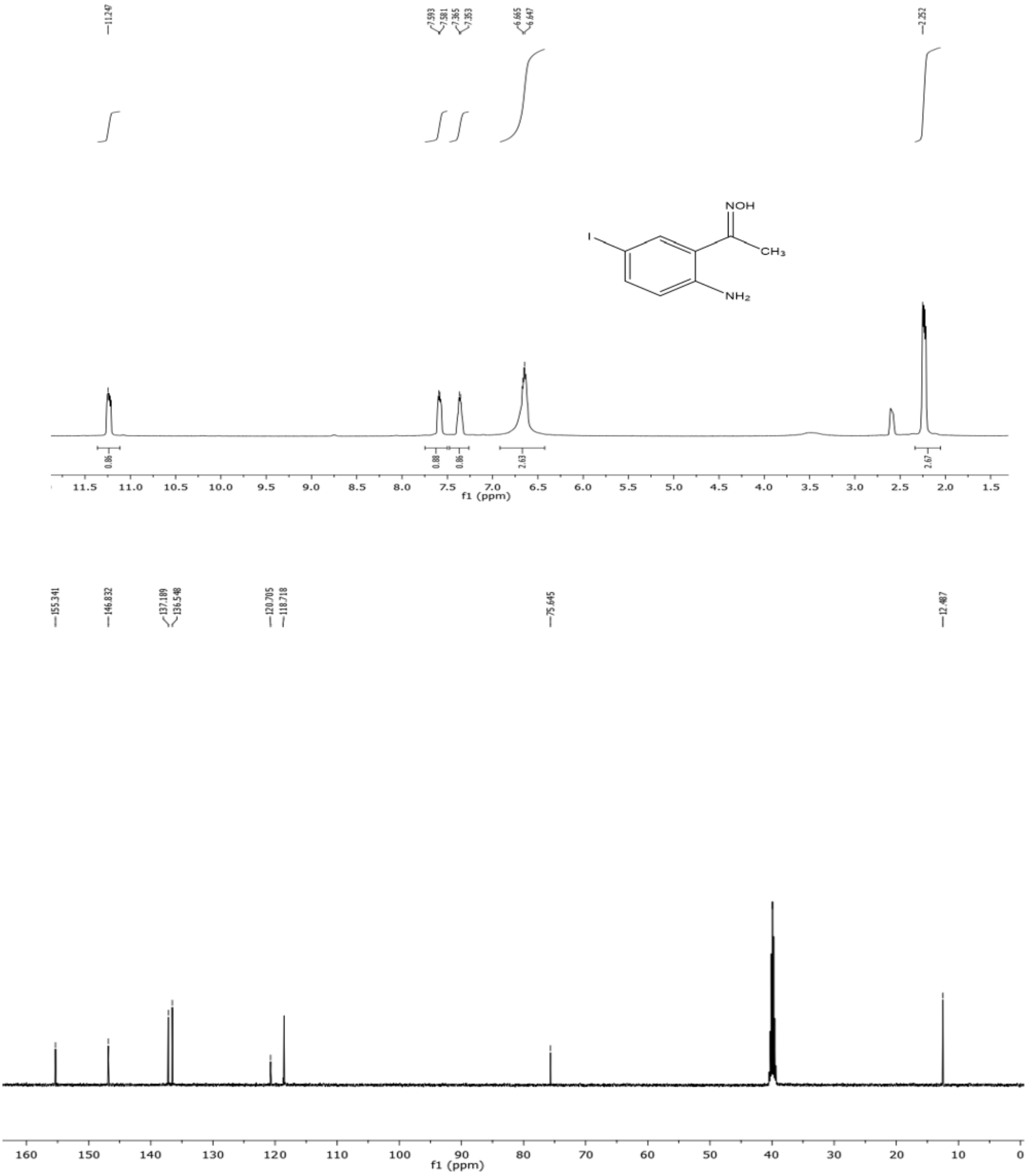

Figure S1.2: ^{1}H- and ^{13}C-NMR spectra of **2b** in DMSO-d_6 at 500 MHz and 125 MHz, respectively.

1-(2-Amino-5-iodophenyl)ethan-1-one oxime (**2b**)

Brown solid (1.78 g, 89%), mp 136–137 °C; ν_{max} (ATR): 818, 1002, 1058, 1278, 1483, 1610, 2983, 3355, 3673 cm^{-1}; ^{1}H-NMR (500 MHz, DMSO-d_6): δ 2.16 (s, 3H, CH_3), 6.12 (s, 2H, NH_2), 7.45 (d, *J* = 1.5 Hz, 1H, H-3), 7.59 (d, *J* = 1.5 Hz, 1H, H-4), 7.35 (s, 1H, H-6), 11.25 (s, 1H, OH); ^{13}C-NMR (125 MHz, DMSO-d_6): δ 12.4, 75.6, 118.6, 120.7, 136.5, 137.2, 146.9, 155.3; HRMS (ES): MH$^+$, calcd for $C_8H_9N_2OI$: 276.9760; found 276.9835.

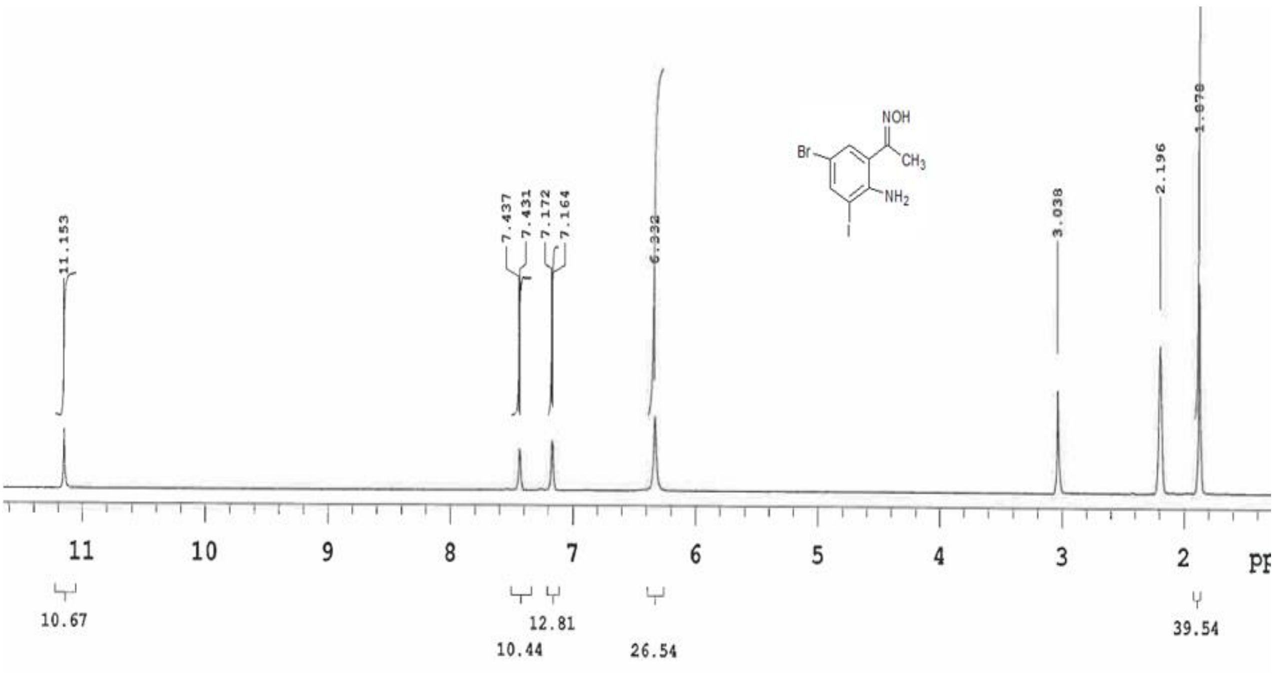

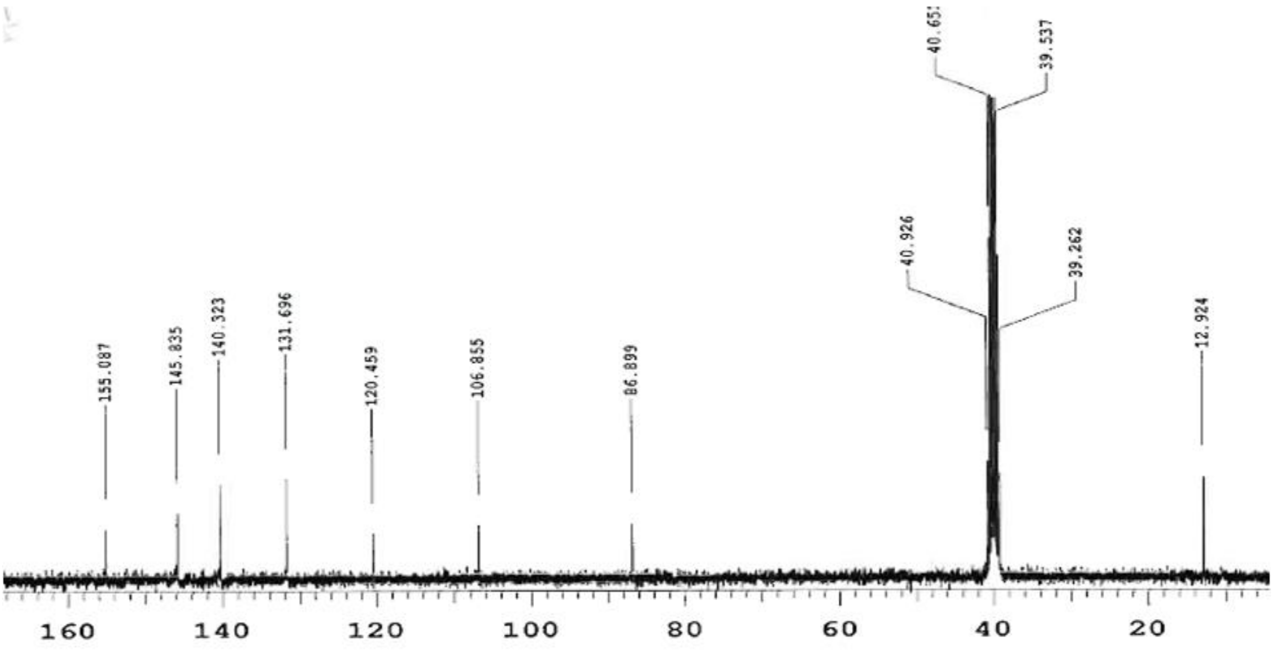

Figure S1.3: ^{1}H- and ^{13}C-NMR spectra of **2c** in DMSO-d_6 at 500 MHz and 125 MHz, respectively.

1-(2-Amino-5-bromo-3-iodophenyl)ethan-1-one oxime (**2c**)

White solid (1.80 g, 90%), mp. 175–176 °C (Lit. 174–176 °C [2]); ν_{max} (ATR): 680, 755, 860, 1002, 1232, 1364, 1425, 1526, 1738, 2833, 3207, 3364 cm^{-1}; ^{1}H-NMR (500 MHz, DMSO-d_6): δ 3.94 (s, 3H, CH_3), 6.24 (s, 2H, -NH_2), 7.09 (s, 1H, H-4), 7.36 (s, 1H, H-6), 11.06 (s, 1H, NH); ^{13}C-NMR (125 MHz, DMSO-d_6): δ 12.9, 86.9, 106.9, 120.5, 131.7, 140.3, 145.8, 155.1.

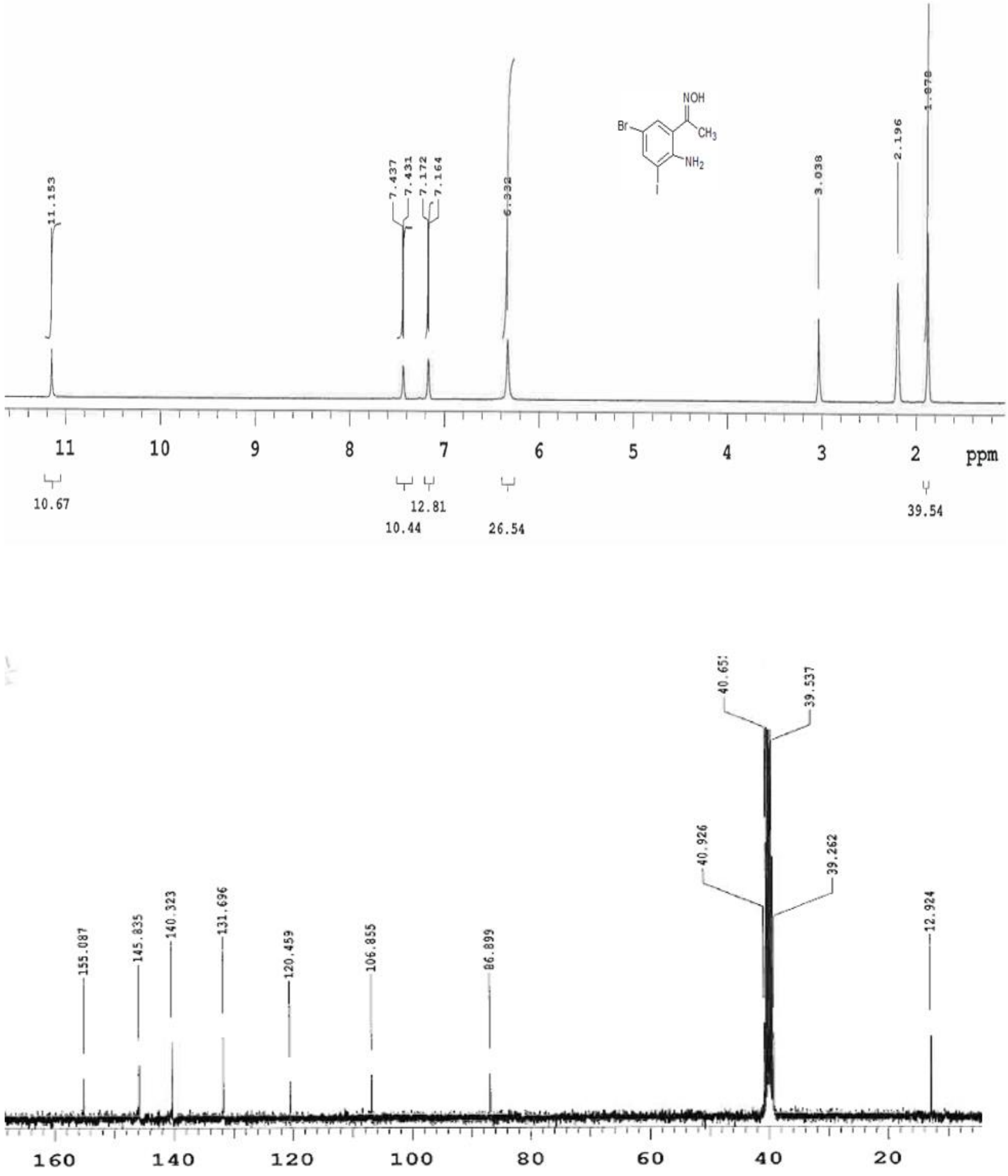

Figure S1.4: ^{1}H- and ^{13}C-NMR spectra of **2d** in DMSO-d_6 at 500 MHz and 125 MHz, respectively.

1-(2-Amino-3-bromo-5-iodophenyl)ethan-1-one oxime (**2d**)

Brown solid (1.78 g, 89%); mp 171–172 °C; ν_{max} (ATR) 862, 1005, 1067, 1233, 1386, 1531, 2990, 3366 cm^{-1}; ^{1}H-NMR (500 MHz, DMSO-d_6): δ 2.16 (s, 3H, CH_3), 6.62 (s, 2H, NH_2), 7.45 (d, J = 1.5 Hz, 1H, H-4), 7.71(d, J = 1.5 Hz, 1H, H-4), 11.44 (s, 1H, OH); ^{13}C-NMR (125 MHz, DMSO-d_6) 12.6, 86.5, 106.9, 120.5, 131.5, 140.3, 145.4, 154.8; HRMS (ES): MH^{+}, calcd for $C_8H_8N_2OBrI$: 354.8865; found 354.8943.

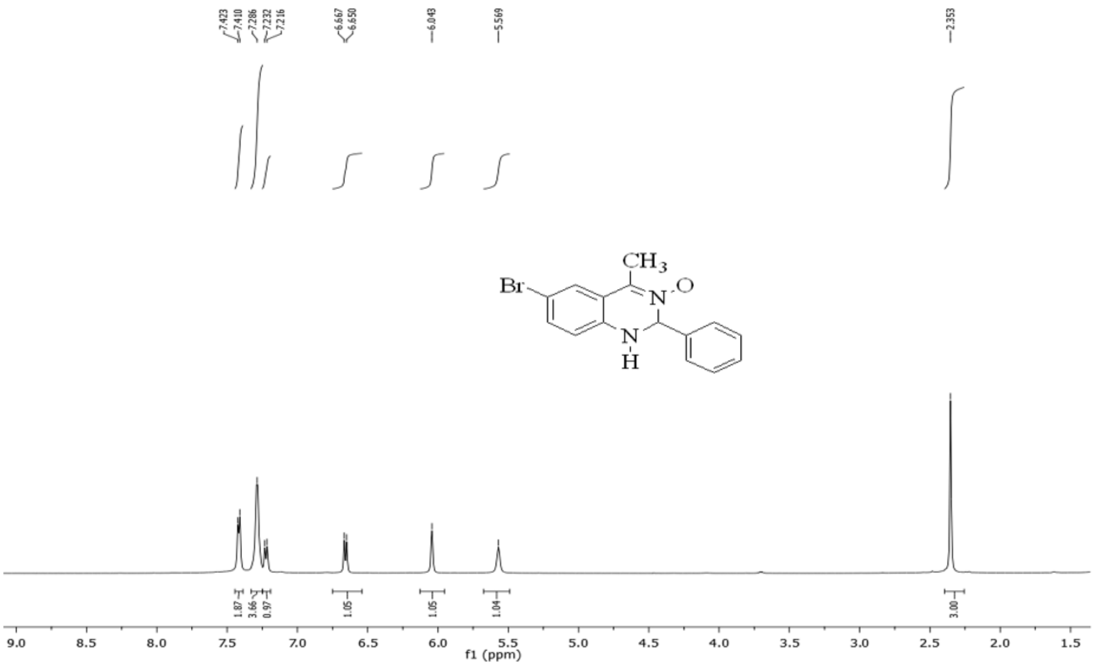

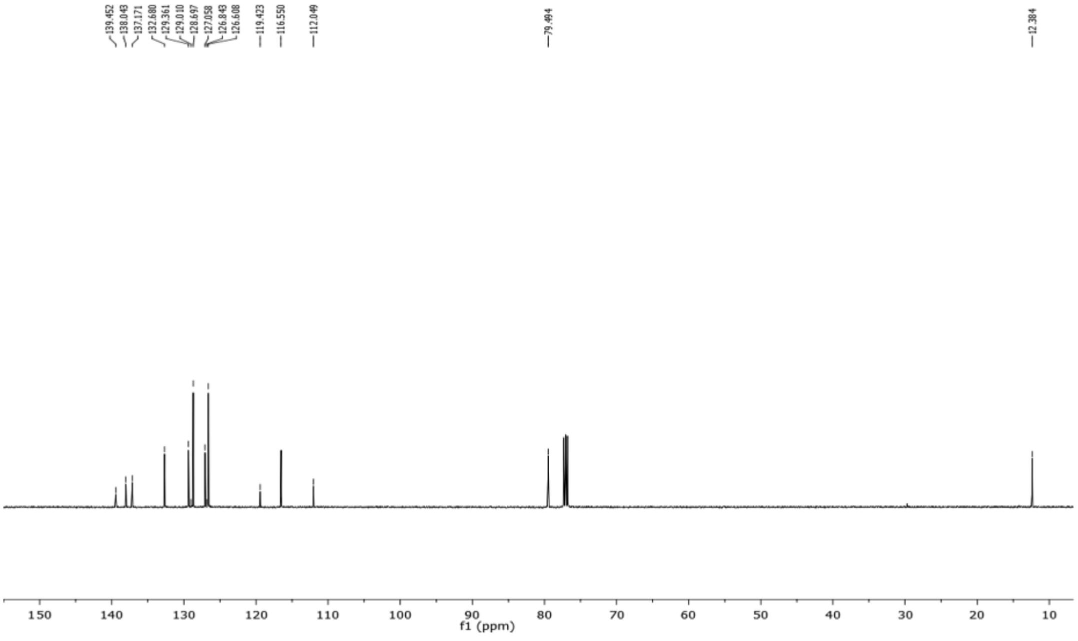

Figure S1.5: ^{1}H- and ^{13}C-NMR spectra of **3a** in $CDCl_3$ at 500 MHz and 125 MHz, respectively.

6-Bromo-4-methyl-2-phenyl-1,2-dihydroquinazoline 3-oxide (**3a**)

Yellow solid (0.30 g, 62%), R_f (5% EtOAc–toluene) 0.64, mp 158–160 °C (Lit. 160–161°C [1]); ν_{max} (ATR) 815, 1072, 1199, 1268, 1407, 1492, 1564, 1600, 2987, 3237 cm^{-1}; ^{1}H-NMR (500 MHz, $CDCl_3$): δ 2.35 (s, 3H, $-CH_3$), 5.57 (s, 1H, H-2), 6.04 (s, 1H, NH), 6.65 (d, J = 8.5 Hz, 1H, H-8), 7.22 (d, J = 8.0 Hz, 1H, H-7), 7.23–7.29 (m, 4H, H-2',6' and H-3',5'), 7.42 (d, J = 6.5 Hz, 2H, H-4' and H-5); ^{13}C-NMR (125 MHz, $CDCl_3$): δ 12.4, 79.5, 112.0, 116.6, 132.0, 119.4, 126.6, 126.8, 127.1, 128.7, 129.0, 129.4, 132.7, 137.2, 138.0, 139.5; HRMS (ES): MH^+, calcd for $C_{15}H_{14}N_2OBr$: 317.0211; found 317.0289.

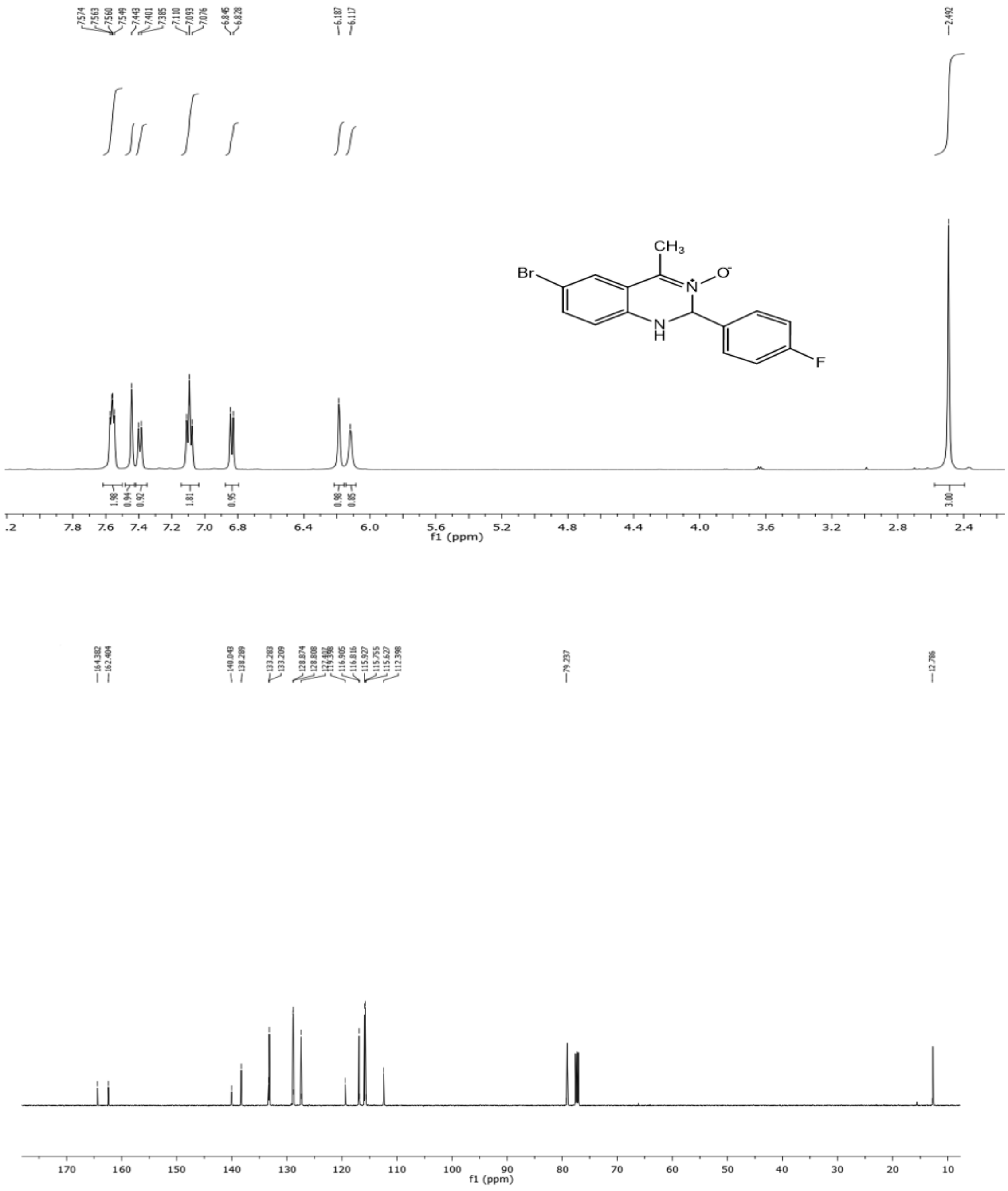

Figure S1.6: ^{1}H- and ^{13}C-NMR spectra of **3b** in $CDCl_3$ at 500 MHz and 125 MHz, respectively.

6-Bromo-2-(4-fluorophenyl)-4-methyl-1,2-dihydroquinazoline 3-oxide (**3b**)

Yellow solid (0.25 g, 50%), R_f (5% EtOAc–toluene) 0.65; mp 183–185 °C; ν_{max} (ATR) 817, 1158, 1197, 1501, 1600, 3001, 3057, 3199, 3234 cm^{-1}; ^{1}H-NMR (500 MHz, $CDCl_3$): δ 2.48 (s, 3H, -CH_3), 6.10 (s, 1H, H-2), 6.19 (s, 1H, NH), 6.83 (d, J = 8.5 Hz, 1H, H-8), 7.10 (t, J = 8.5 Hz, 2H, H-3',5'), 7.40 (d, J = 8.0 Hz, 1H, H-7), 7.44 (s, 1H, H-5), 7.57 (dd, J_{HH} = 5.5 Hz and J_{HH} = 7.0 Hz, 2H, H-2',6'); ^{13}C-NMR (125 MHz, $CDCl_3$): δ 12.9, 79.2, 112.4, 115.6 (d, $^{2}J_{CF}$ = 21.5 Hz), 116.8, 116.9, 119.4, 127.4, 128.8 (d, $^{3}J_{CF}$ = 8.3 Hz), 130.9, 132.8 (d, $^{2}J_{CF}$ = 2.7 Hz), 135.3, 138.3, 140.0, 162.4 (d, $^{2}J_{CF}$ = 247.3); HRMS (ES): MH^{+}, calcd for $C_{15}H_{12}N_2OBrF$: 335.0117; found 335.0200.

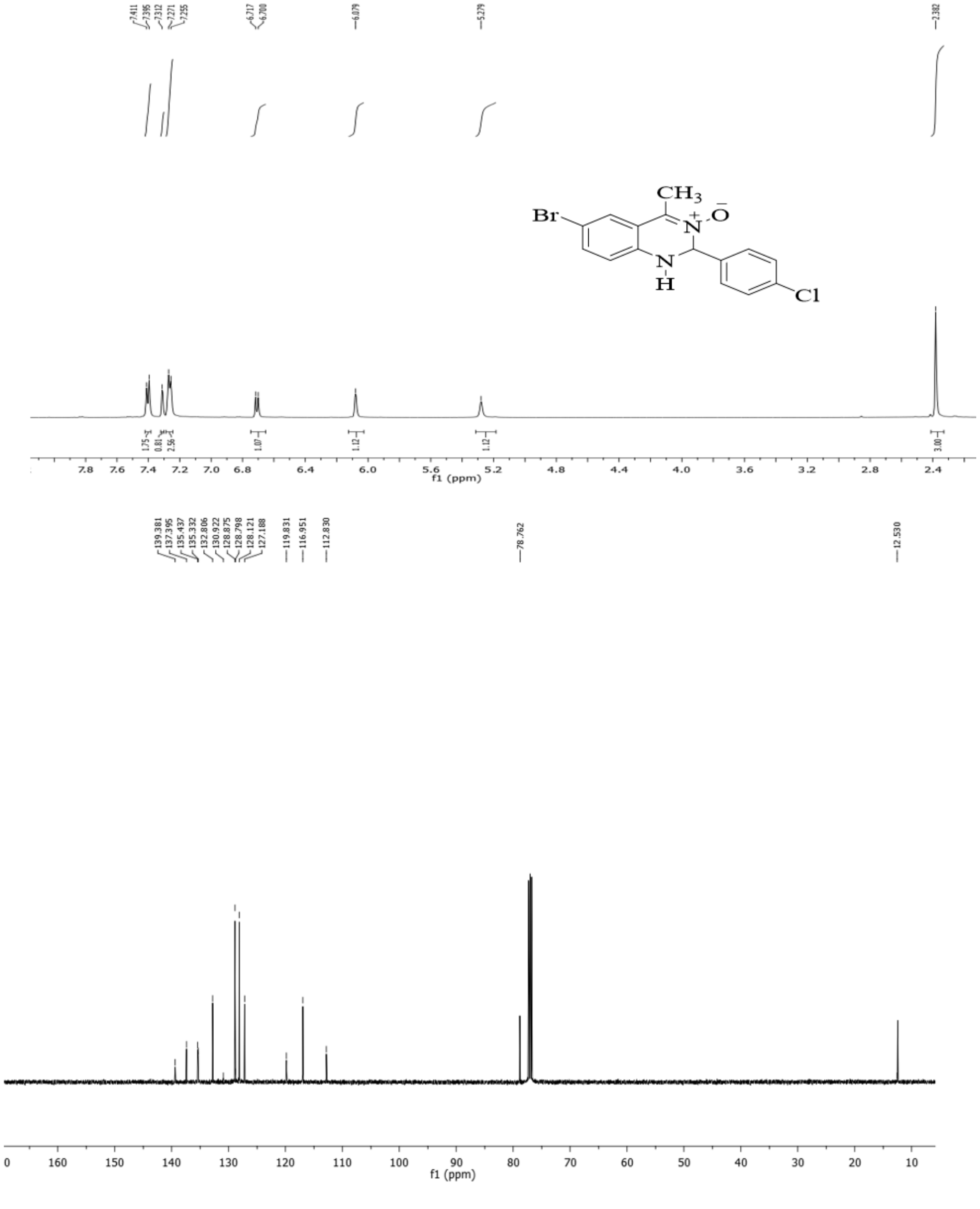

Figure S1.7: ^{1}H- and ^{13}C-NMR spectra of **3c** in $CDCl_3$ at 500 MHz and 125 MHz, respectively.

6-Bromo-2-(4-chlorophenyl)-4-methyl-1,2-dihydroquinazoline 3-oxide (**3c**)

Yellow solid (0.42 g, 84%), R_f (5% EtOAc–toluene) 0.66, mp. 171–173 °C (Lit. 170–171 °C [1]); ν_{max} (ATR) 822, 1016, 1093, 1194, 1291, 1489, 1543, 1598, 3271 cm^{-1}; ^{1}H-NMR (500 MHz, $CDCl_3$): δ 2.38 (s, 3H, CH_3), 5.28 (s, 1H, H-2), 6.08 (1H, s ,NH), 6.70 (d, *J* = 8.7 Hz, 1H, H-8), 7.26 (d, *J* = 8.0 Hz, 3H, H-2',6' and H-7), 7.30 (s, 1H, H-5), 7.40 (d, *J* = 8.0 Hz, 2H, H-3',5'); ^{13}C-NMR (125 MHz, $CDCl_3$): δ 12.5, 78.8, 112.8, 116.9, 119.8, 127.2, 128.1, 128.8, 128.9, 130.9, 132.8, 135.3, 135.4, 137.4, 139.4; HRMS (ES): MH$^+$, calcd for $C_{15}H_{13}N_2OBrCl$: 350.9822; found 350.9886.

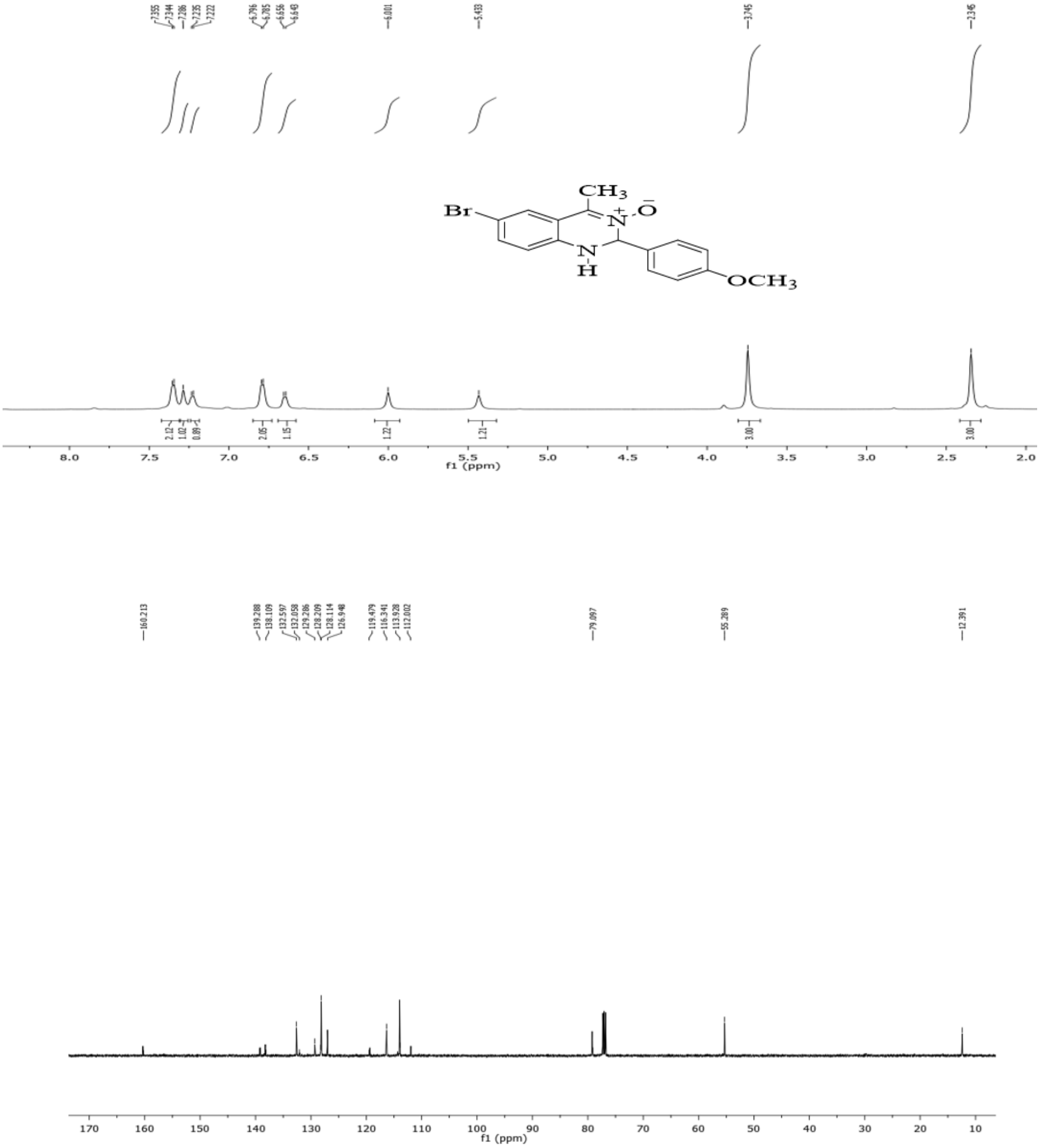

Figure S1.8: ^{1}H- and ^{13}C-NMR spectra of **3d** in $CDCl_3$ at 500 MHz and 125 MHz, respectively.

6-Bromo-2-(4-methoxyphenyl)-4-methyl-1,2-dihydroquinazoline 3-oxide (**3d**)

Yellow solid (0.45 g, 90%), R_f (5% EtOAc–toluene) 0.62, mp 194–196 °C; ν_{max} (ATR) 825, 1028, 1199, 1291, 1469, 1517, 1600, 2960, 3281 cm^{-1}; ^{1}H-NMR (500 MHz, $CDCl_3$): δ 2.35 (s, 3H, -CH_3), 5.43 (s, 1H, H-2), 6.00 (s 1H, NH), 6.65 (1H, d, *J* = 6.5 Hz, H-8), 6.79 (2H, d, *J* = 5.5 Hz, H-3',5'), 7.22 (d, *J* = 6.5 Hz,1H, H-7), 7.35 (d, *J* = 5.5 Hz, 2H, H-2',6'); ^{13}C-NMR (125 MHz, $CDCl_3$): δ 12.4, 55.3, 79.1, 112.0, 113.9, 116.3, 119.5, 126.9, 128.1, 128.2, 129.3, 132.0, 132.6, 138.1, 139.3, 160.2; HRMS (ES): MH^+, calcd for $C_{16}H_{16}N_2O_2Br$: 346.0317; found 347.0391.

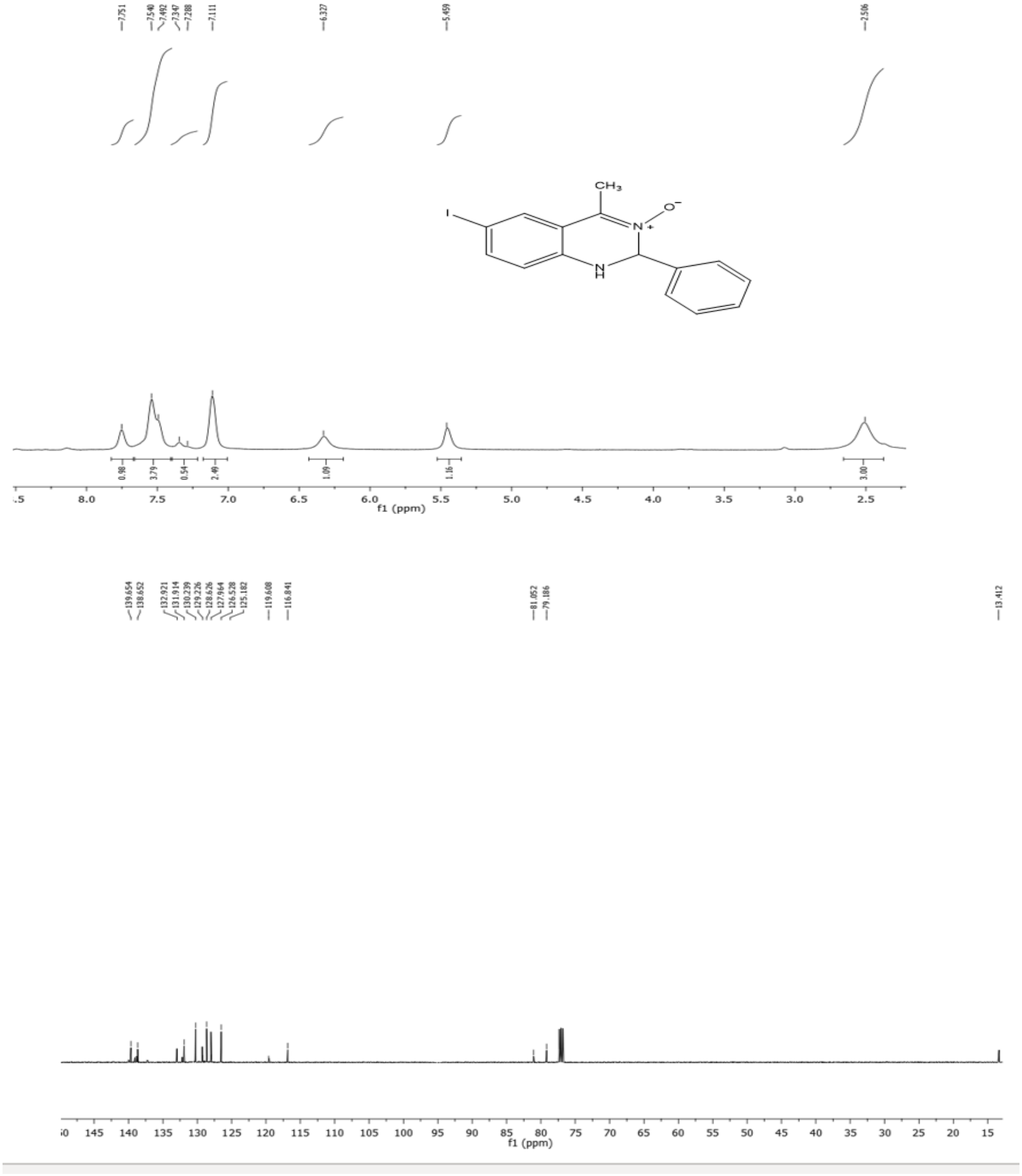

Figure S1.9: ^{1}H- and ^{13}C-NMR spectra of **3e** in $CDCl_3$ at 500 MHz and 125 MHz, respectively.

6-Iodo-4-methyl-2-phenyl-1,2-dihydroquinazoline 3-oxide (**3e**)

Yellow solid (0.34 g, 68%), R_f (5% EtOAc–toluene) 0.63, mp 184–186 °C; ν_{max} (ATR) 815, 1028, 1180, 1268, 1489, 1561, 1599, 2987, 3156, 3228 cm^{-1}; ^{1}H-NMR (500 MHz, $CDCl_3$): δ 2.52 (s, 3H, $-CH_3$), 5.36 (s, 1H, H-2), 6.27 (s, 1H, NH), 6.70 (d, 1H, J = 6.5 Hz, H-8), 7.44 (m, 3H, H-4' and H-2',6'), 7.55–7.59 (m, 4H, H-3',5', H-5 and H-7); ^{13}C-NMR (125 MHz, $CDCl_3$) δ 13.4, 79.2, 81.1, 116.8, 119.6, 125.2, 126.5, 127.9, 128.6, 129.2, 130.2, 131.9, 132.9, 138.7, 139.7; HRMS (ES^+): m/z $[M + H]^+$ calcd for $C_{15}H_{14}N_2OI$: 365.0073; found 365.0149.

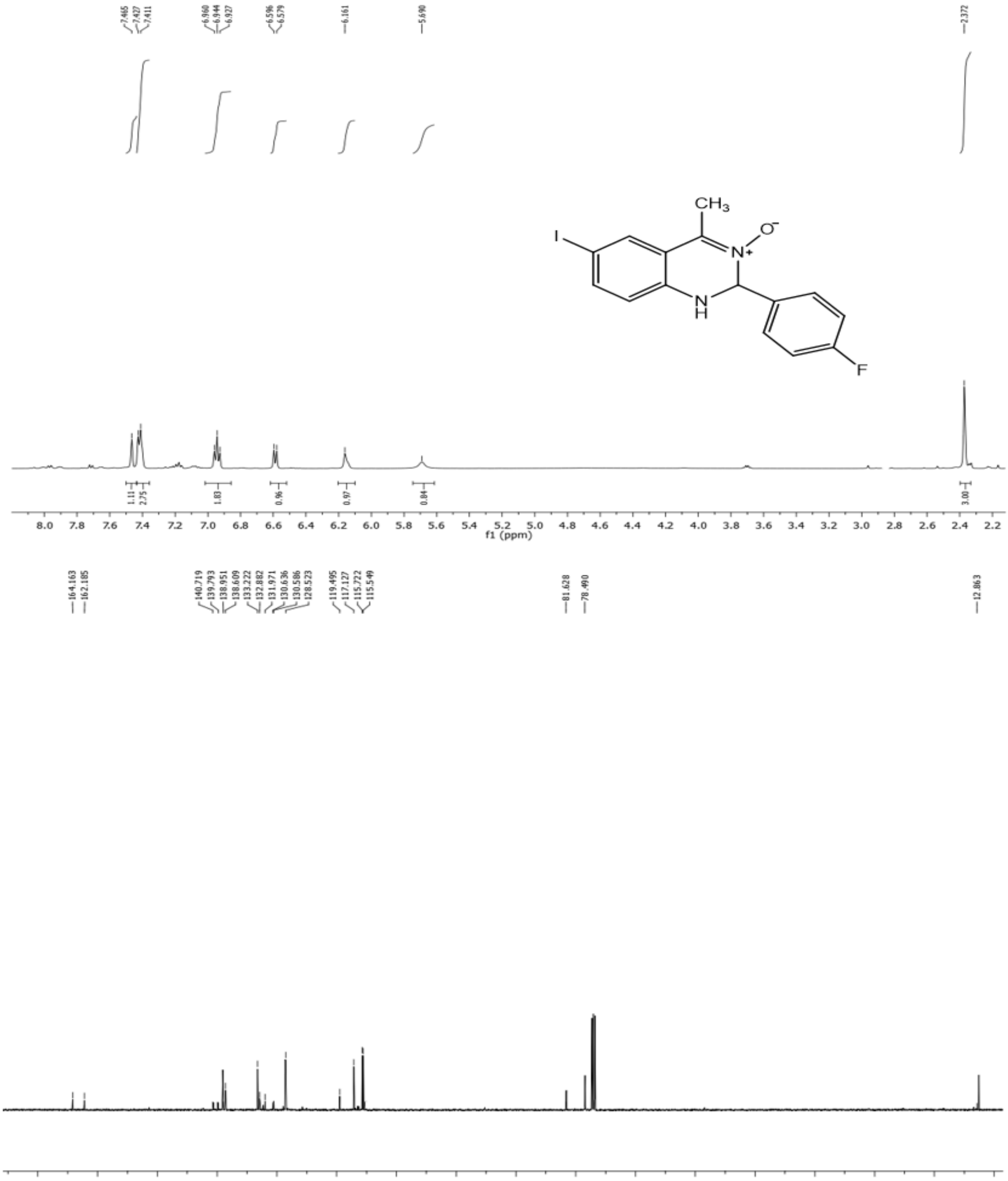

Figure S1.10: ^{1}H- and ^{13}C-NMR spectra of **3f** in $CDCl_3$ at 500 MHz and 125 MHz, respectively.

2-(4-Fluorophenyl)-6-iodo-4-methyl-1,2-dihydroquinazoline 3-oxide (**3f**)

Yellow solid (0.36 g, 72%), R_f (5% EtOAc–toluene) 0.64; mp 180–182 °C; ν_{max} (ATR) 813, 1083, 1185, 1286, 1489, 1506, 1596, 1603, 2902, 2974, 3216 cm^{-1}; ^{1}H-NMR (500 MHz, $CDCl_3$): δ 2.37 (s, 3H, -CH_3), 5.67 (s, 1H, H-2), 6.16 (s, 1H, NH), 6.56 (d, J = 8.5 Hz, 1H, H-8), 6.94 (t, J = 8.5 Hz, 2H, H-3',5'), 7.41 (d, J = 8.0 Hz, 3H, H-2',6' and H-7), 7.47 (s, 1H, H-5); ^{13}C-NMR (125 MHz, $CDCl_3$): δ 12.9, 78.5, 81.6, 115.5 (d, $^{2}J_{CF}$ = 21.6 Hz), 117.2, 119.5, 128.5, 130.6 (d, $^{3}J_{CF}$ = 8.2 Hz), 131.9, 132.8 (d, $^{2}J_{CF}$ = 2.7 Hz), 133.2, 138.6, 138.9, 140.7, 163.2 (d, $^{2}J_{CF}$ = 247.3 Hz); HRMS (ES): MH^{+}, calcd for $C_{15}H_{12}N_2OFI$: 382.9978; found 382.9811.

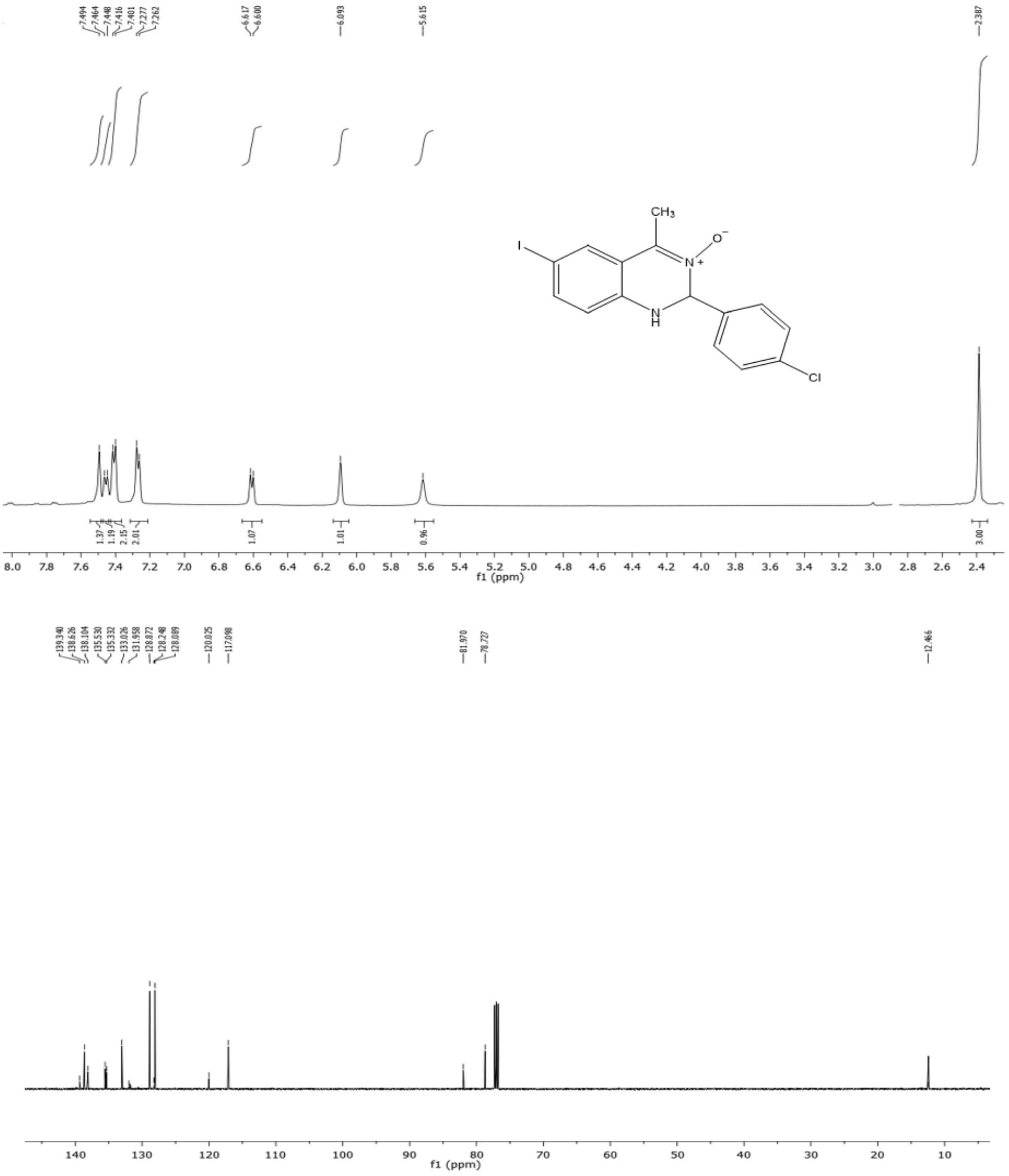

Fig. S1.11: ^{1}H- and ^{13}C-NMR spectra of **3g** in $CDCl_3$ at 500 MHz and 125 MHz, respectively.

6-Iodo-2-(4-Chlorophenyl)-6-iodo-4-methyl-1,2-dihydroquinazoline 3-oxide (**3g**)

Yellow solid (0.44 g, 88%), R_f (5% EtOAc–toluene) 0.65, mp182–184 °C; ν_{max} (ATR) 818, 1012, 1092, 1199, 1272, 1404, 1485, 1564, 1601, 2987, 3225 cm^{-1}; ^{1}H-NMR (500 MHz, $CDCl_3$): δ 2.39 (s, 3H, -CH_3), 5.62 (s, 1H, H-2), 6.01 (s, 1H, NH), 6.61 (d, *J* = 8.5 Hz, 1H, H-8), 7.26 (d, *J* = 7.5 Hz, 2H, H-2',6'), 7.40 (d, *J* = 7.5 Hz, 2H, H-3',5'), 7.45 (d, *J* = 8.0 Hz, 1H, H-7), 7.49 (s, 1H, H-5); ^{13}C-NMR (125 MHz, $CDCl_3$): δ 12.6, 78.7, 82.1, 117.3, 119.8, 128.2, 129.1, 130.8, 132.1, 133.1, 134.4, 135.7 138.2, 139.1, 139.8; HRMS (ES): MH^{+}, calcd for $C_{15}H_{13}N_2OClI$: 397.9683; found 398.9760.

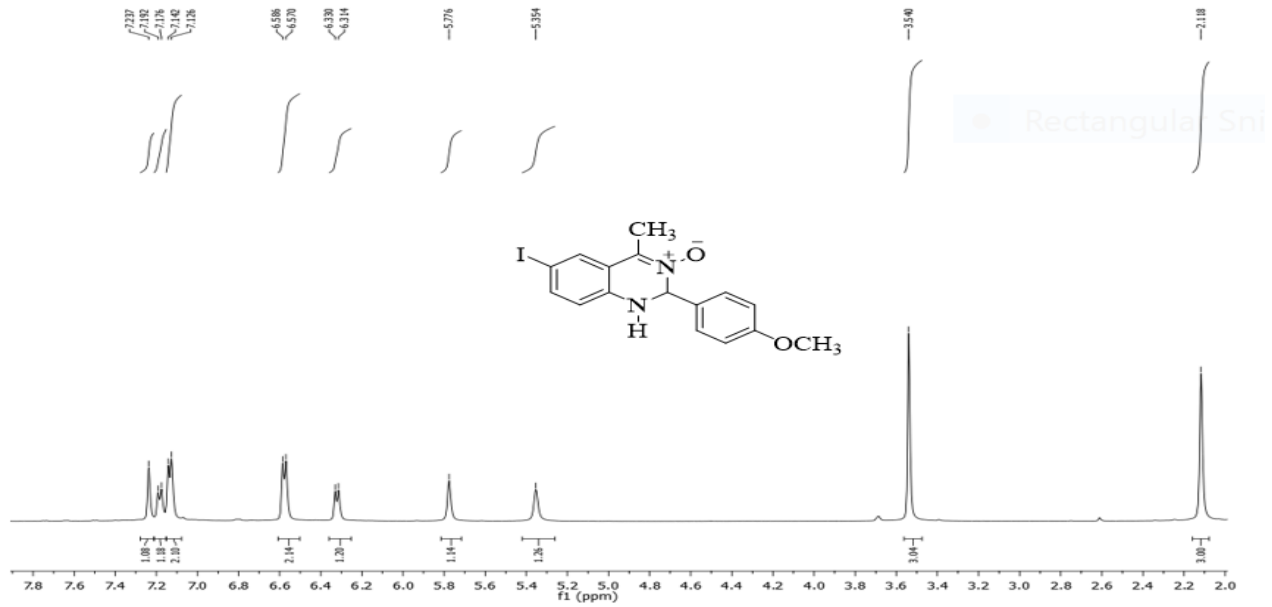

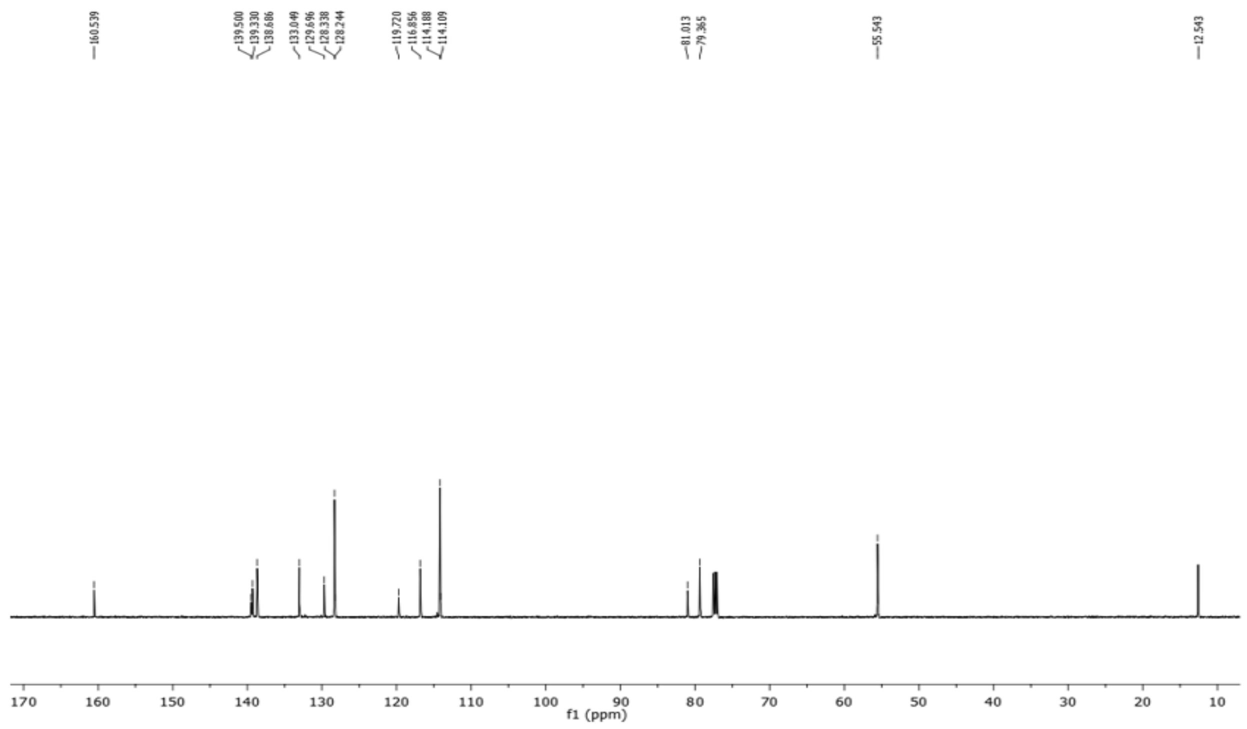

Fig. S1.12: ^{1}H- and ^{13}C-NMR spectra of **3h** in $CDCl_3$ at 500 MHz and 125 MHz, respectively.

6-Iodo-2-(4-methoxyphenyl)-4-methyl-1,2-dihydroquinazoline 3-oxide (**3h**)

Yellow solid (0.43 g, 86%), R_f (5% EtOAc–toluene) 0.60, mp. 176–179 °C; ν_{max} (ATR) 822, 1030, 1203, 1293, 1459, 1515, 1596 (C=N), 2974, 3269 (NH) cm^{-1}; ^{1}H-NMR (500 MHz, $CDCl_3$): δ 2.18 (s, 3H, -CH_3), 3.54 (s, 3H, -OCH_3), 5.35 (s, 1H, H-2), 5.78 (s, 1H, NH), 6.31 (d, J = 8.0 Hz, 1H, H-8), 6.57 (d, J = 8.0 Hz, 2H, H-3',5'), 7.13 (d, J = 8.0 Hz, 2H, H-2',6'), 7.18 (d, J = 8.0 Hz, 1H, H-7), 7.24 (s, 1H, H-5); ^{13}C-NMR (125 MHz, $CDCl_3$): δ 12.5, 55.5, 79.4, 81.0, 114.1, 114.2, 116.8, 119.7, 128.2, 128.3, 129.7, 133.0, 138.7, 139.3, 139.5, 160.5; HRMS (ES): MH^+, calcd for $C_{16}H_{16}N_2O_2I$: 395.0178; found 395.9600.

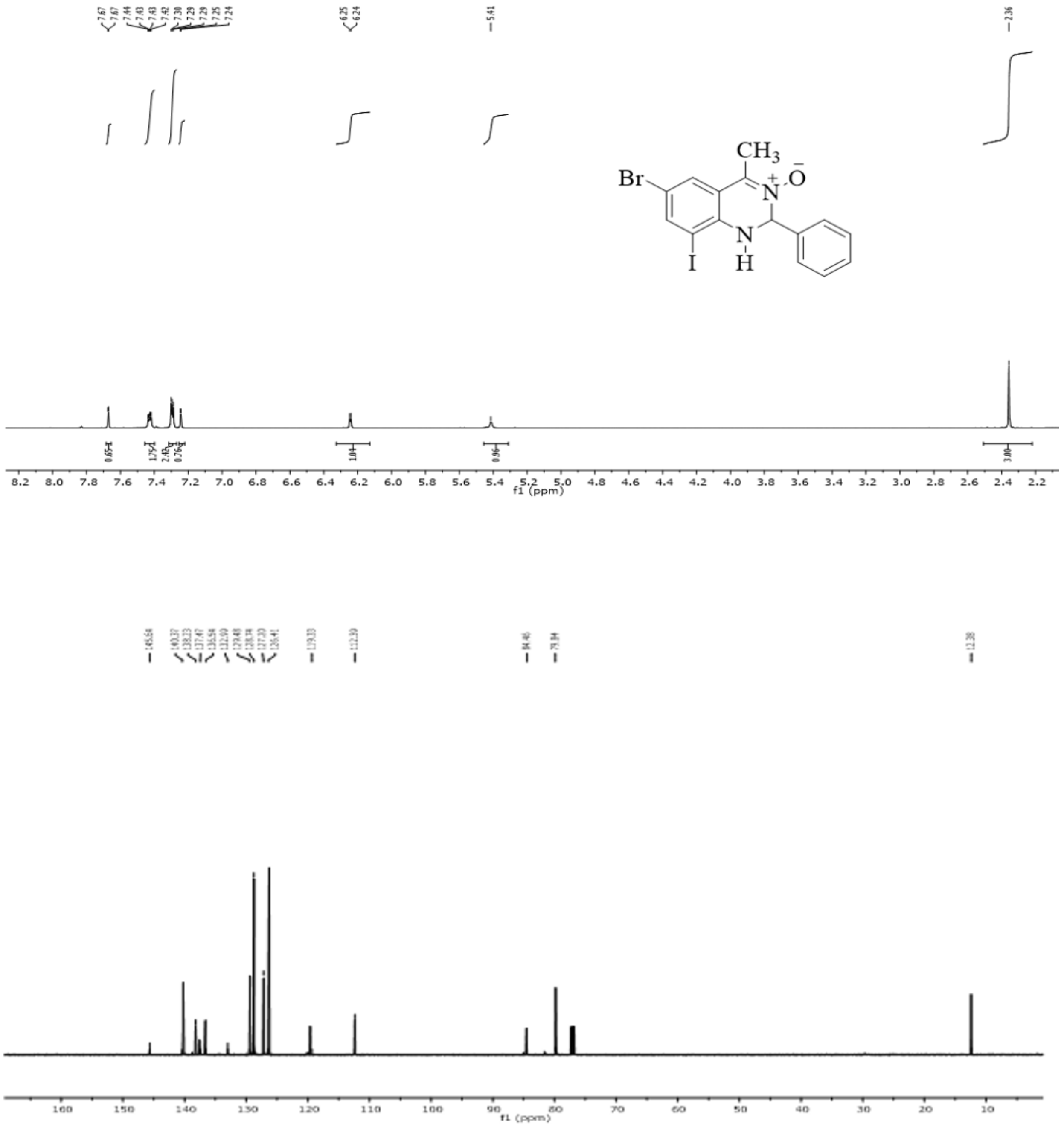

Fig. S1.13: ^{1}H- and ^{13}C-NMR spectra of **3i** in $CDCl_3$ at 500 MHz and 125 MHz, respectively.

6-Bromo-8-iodo-4-methyl-2-phenyl-1,2-dihydroquinazoline 3-oxide (**3i**)

Yellow solid (0.37 g, 74%); R_f (5% EtOAc–toluene) 0.64, mp. 162–164 °C; ν_{max} (ATR); 1069, 1231, 1262, 1327, 1478, 1584, 1600, 2921, 3288.3 cm^{-1}; ^{1}H-NMR (500 MHz, $CDCl_3$): δ 2.36 (s, 3H, -CH_3), 5.41 (s, 1H, H-2), 6.24 (d, *J* = 3.5 Hz, 1H, NH), 7.24 (d, *J* = 2.0 Hz, 1H, H-5), 7.25–7.30 (m, 3H, H-4′ and H-2′,6′), 7.40–7.44 (m, 2H, H-3′,5′), 7.67 (d, *J* = 2.0 Hz, 1H, H-7); ^{13}C-NMR (125 MHz, $CDCl_3$): δ 12.4, 79.8, 84.5, 112.3, 119.7, 126.2, 127.2, 128.7, 129.4, 132.9, 136.6, 137.6, 138.3, 140.3, 145.6; HRMS (ES): MH^{+}, calcd for $C_{15}H_{12}N_2OBrI$: 442.9178; found 442.9246.

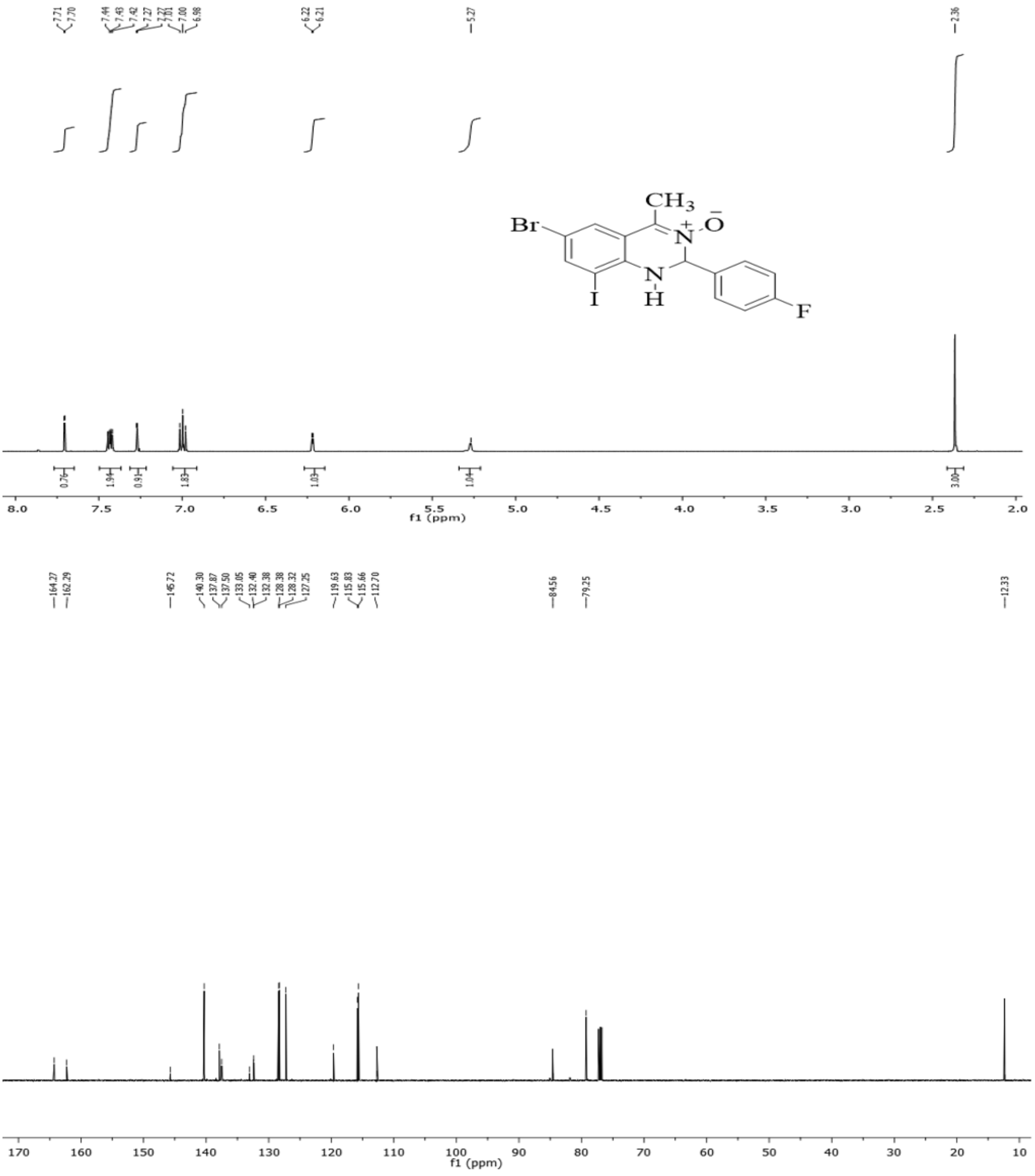

Fig. S1.14: ^{1}H- and ^{13}C-NMR spectra of **3j** in $CDCl_3$ at 500 MHz and 125 MHz, respectively.

6-Bromo-2-(4-fluorophenyl)-8-iodo-4-methyl-1,2-dihydroquinazoline 3-oxide (**3j**)

Yellow solid (0.30 g, 60%); R_f (5% EtOAc–toluene) 0.66, mp. 170–173 °C; ν_{max} (ATR) 1010, 1222, 1279, 1404, 1479, 1503, 1602, 2923, 3236, ^{1}H-NMR (500 MHz, $CDCl_3$): δ 2.37 (s, 3H, -CH_3), 5.27 (s, 1H, H-2), 6.22 (d, J = 3.5 Hz, 1H, NH), 6.99 (t, J = 8.5 Hz, 2H, H-3′,5′), 7.27 (d, J = 2.0 Hz, 1H, H-5), 7.43 (dt, J_{HH} = 8.5 Hz and J_{HF} = 5.5 Hz, 2H, H-2′,6′), 7.71 (d, J = 2.0 Hz, 1H, H-7); ^{13}C-NMR (125 MHz, $CDCl_2$): δ 12.3, 79.3, 84.6, 112.7, 115.7 (d, $^2J_{CF}$ = 21.3 Hz), 119.6, 127.3, 128.4 (d, $^3J_{CF}$ = 7.7 Hz), 132.4 (d, $^4J_{CF}$ = 2.5 Hz), 133.1, 137.5, 137.9, 140.3, 145.7, 162.3 (d, $^1J_{CF}$ = 247.5 Hz); HRMS (ES): MH^+, calcd for $C_{15}H_{11}N_2OBrFI$: 460.9083; found 460.8727.

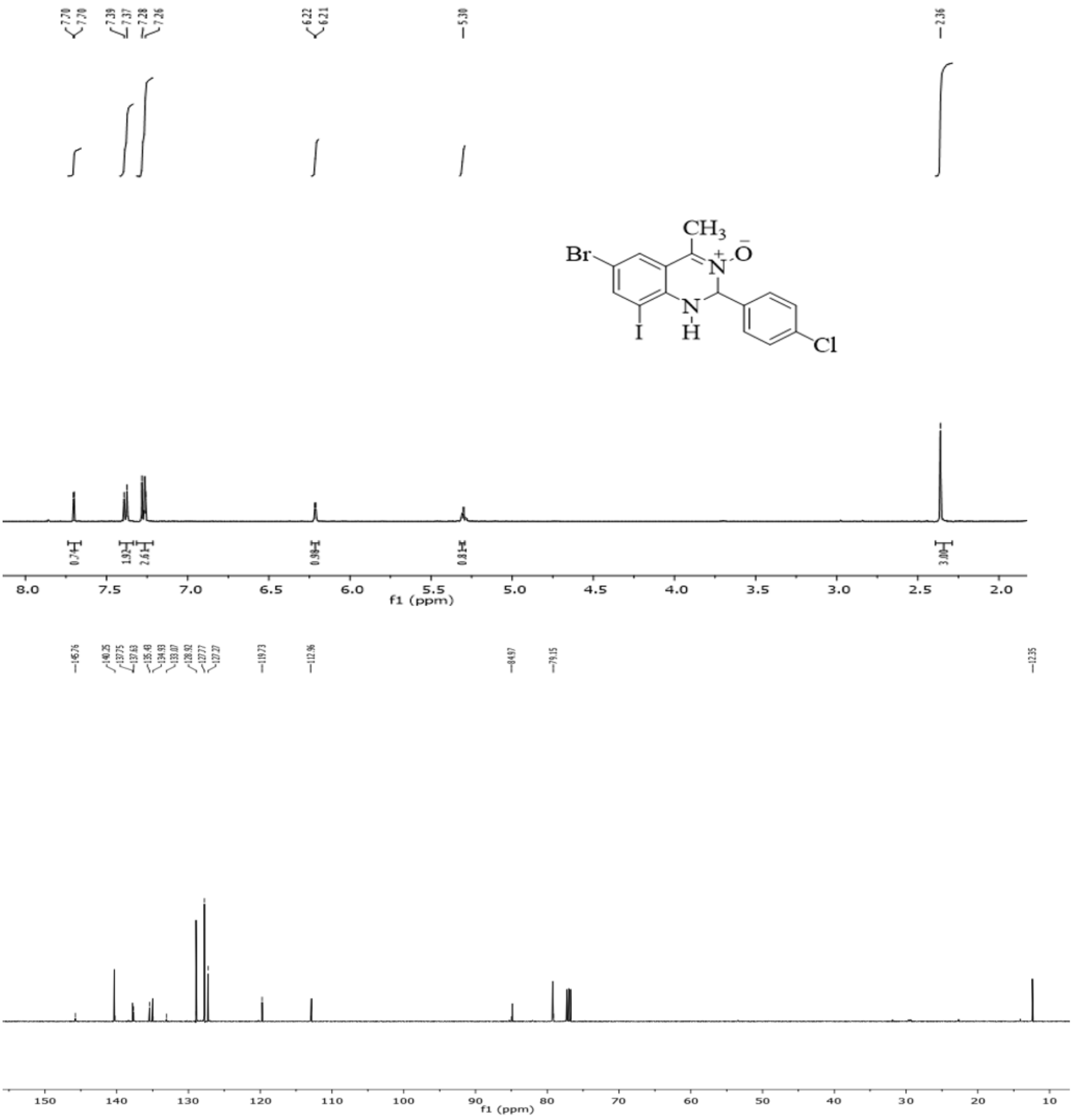

Fig. S1.15: ^{1}H- and ^{13}C-NMR spectra of **3k** in $CDCl_3$ at 500 MHz and 125 MHz, respectively.

6-Bromo-2-(4-chlorophenyl)-8-iodo-4-methyl-1,2-dihydroquinazoline 3-oxide (**3k**)

Yellow solid (0.40 g, 80%); R_f (5% EtOAc–toluene) 0.70, mp. 168-170 °C; ν_{max} (ATR) 1172, 1228, 1261, 1399, 1481, 1575, 2916, 2972, 3230; ^{1}H-NMR (500 MHz, $CDCl_3$): δ 2.36 (s, 3H, -CH_3), 5.29 (s, 1H, H-2), 6.21 (d, *J* = 3.5 Hz, 1H, NH), 7.26 (t, *J* = 2.5 Hz, 2H, H-2′,6′), 7.27 (d, *J* = 2.0 Hz, 1H, H-5), 7.28 (d, *J* = 8.0 Hz, 2H, H-3′,5′), 7.70 (d, *J* = 2.0 Hz, 1H, H-7); ^{13}C-NMR (125 MHz, $CDCl_3$): δ 12.4, 79.2, 85.0, 113.0, 119.7, 127.3, 127.3, 127.8, 128.9, 133.1, 134.9, 135.4, 137.6, 137.8, 140.3, 145.8; HRMS (ES): MH^+, calcd for $C_{16}H_{14}N_2O_2BrI$: 472.9283; found 472.9355.

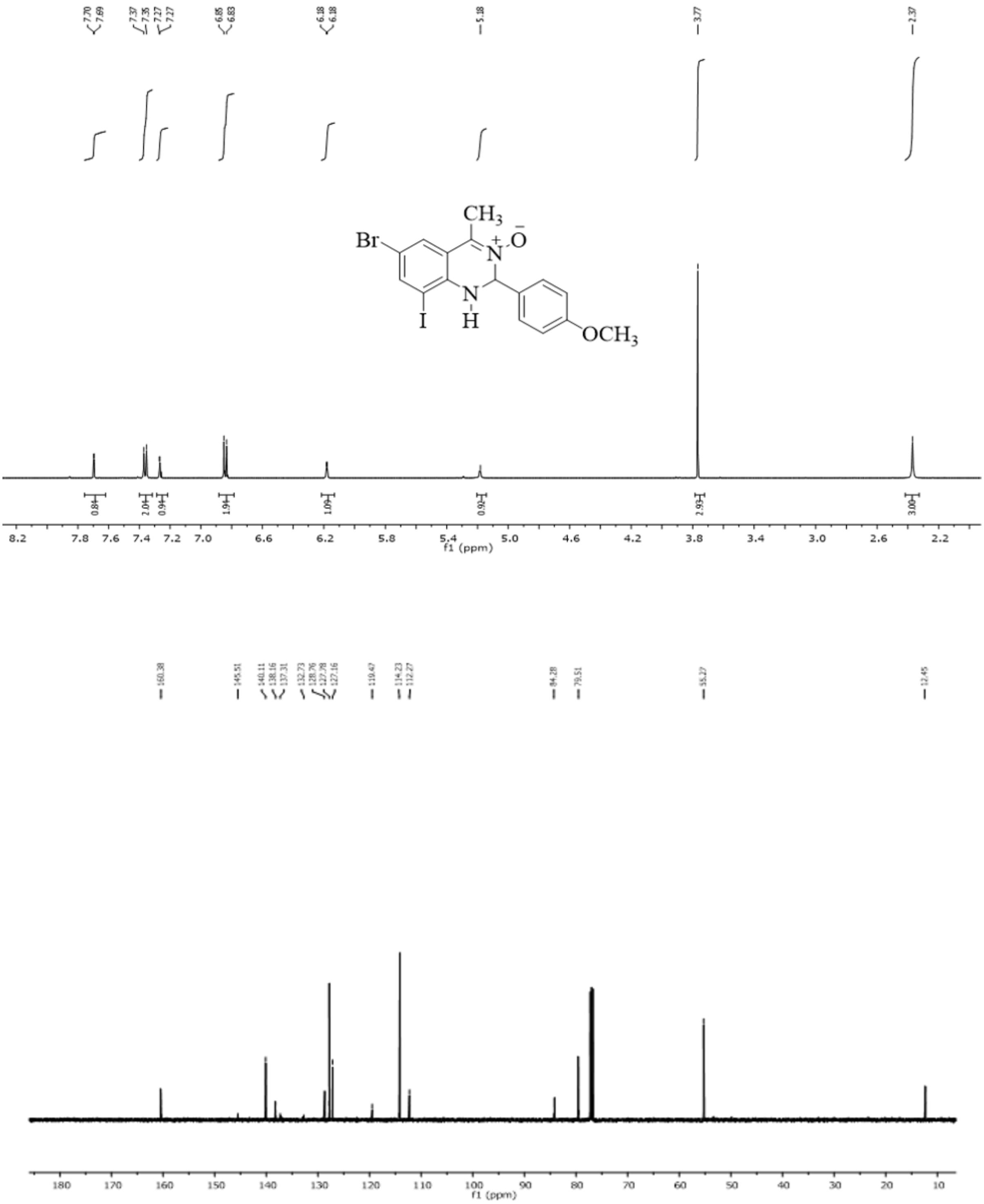

Fig. S1.16: ^{1}H- and ^{13}C-NMR spectra of **3l** in $CDCl_3$ at 500 MHz and 125 MHz, respectively.

6-Bromo-8-iodo-2-(4-methoxyphenyl)-4-methyl-1,2-dihydroquinazoline 3-oxide (**3l**)

Yellow solid (0.42 g, 84%), R_f (5% EtOAc–toluene) 0.63, mp. 153–155 °C; ν_{max} (ATR); 1024, 1168, 1230, 1482, 1503, 1575, 1606, 2968, 3223 cm^{-1}; ^{1}H-NMR (500 MHz, $CDCl_3$): δ 2.37 (s, 3H, -CH_3), 3.77 (s, 3H, -OCH_3), 5.18 (s, 1H, H-2), 6.18 (d, *J* = 3.0 Hz, 1H, NH), 6.84 (d, *J* = 9.0 Hz, 2H, H-2′,6′), 7.27 (d, *J* = 2.0 Hz, 1H, H-5), 7.36 (d, *J* = 8.5 Hz, 2H, H-3′,5′), 7.70 (d, *J* = 2.0 Hz, 1H, H-7); ^{13}C-NMR (125 MHz, $CDCl_3$): δ 12.5, 55.4, 79.5, 84.3, 112.3, 114.2, 119.5, 127.2, 127.4, 128.4, 132.7, 137.3, 138.2, 140.1, 145.5, 160.4; HRMS (ES): MH^{+}, calcd for $C_{16}H_{14}N_2O_2BrI$: 472.9283; found 472.9355.

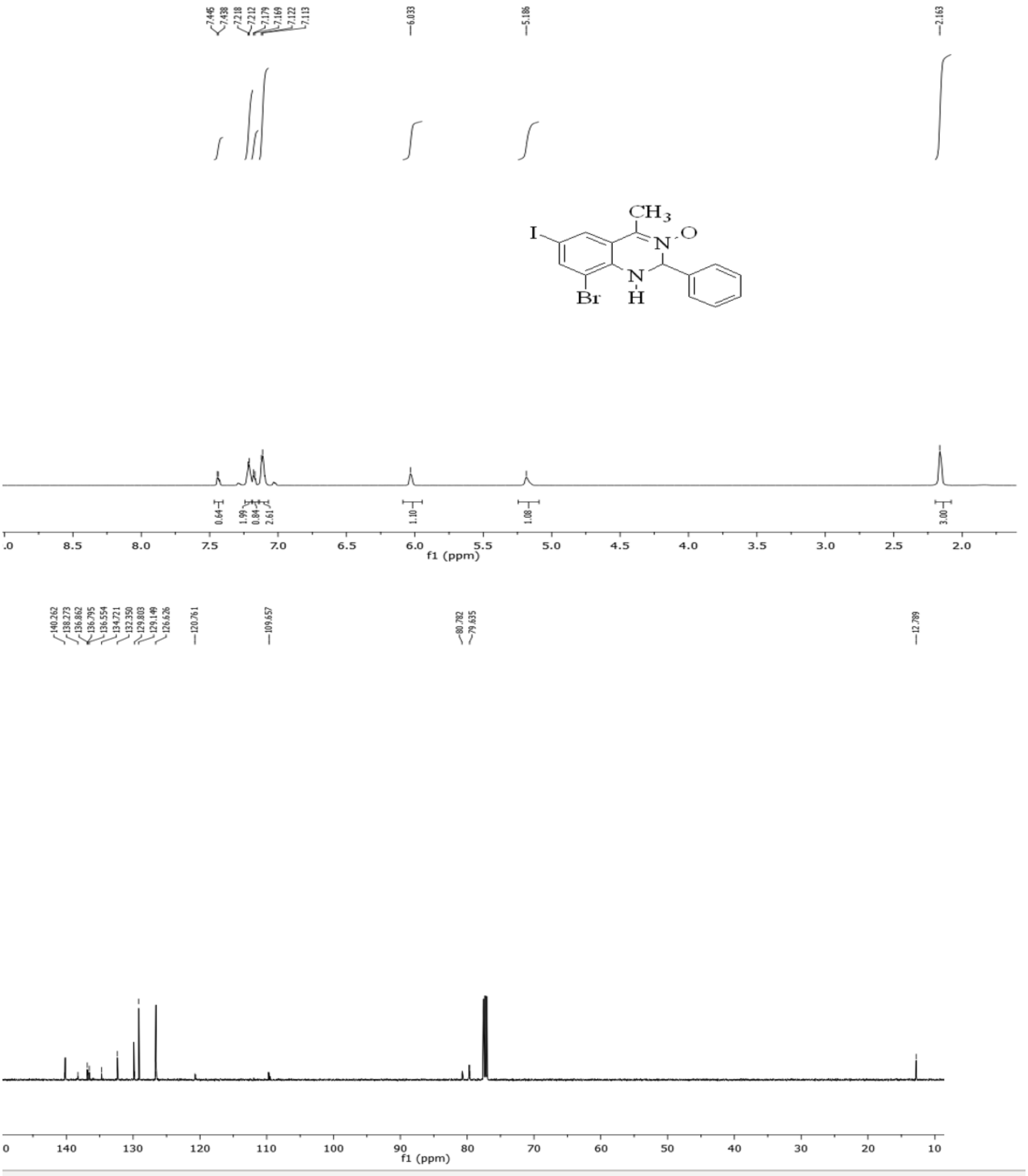

Fig. S1.17: ^{1}H- and ^{13}C-NMR spectra of **3m** in $CDCl_3$ at 500 MHz and 125 MHz, respectively.

8-Bromo-6-iodo-4-methyl-2-phenyl-1,2-dihydroquinazoline 3-oxide (**3m**)

Yellow solid (0.38 g, 76%), R_f (5% EtOAc–toluene) 0.62, mp. 180–181 °C; ν_{max} (ATR) 856, 1082, 1184, 1234, 1446, 1485, 1589, 2920, 3250 cm^{-1}; ^{1}H-NMR (500 MHz, $CDCl_3$): δ 2.16 (s, 3H, -CH_3), 5.19 (s, 1H, H-2), 6.03 (s, 1H, NH), 7.12 (d, *J* = 4.5 Hz, 3H, H-4′ and H-2′,6′), 7.18 (d, *J* = 3.5 Hz, 1H, H-5), 7.21 (d, *J* = 3.5 Hz, 2H, H-7); ^{13}CNMR (125 MHz, $CDCl_3$): δ 12.7, 79.6, 80.9, 109.8, 120.6, 126.7, 129.1, 129.9, 132.4, 134.7, 136.6, 136.8, 138.3, 140.3; HRMS (ES): MH^{+}, calcd for $C_{15}H_{13}N_2OBrI$: 442.9178; found 442.9252.

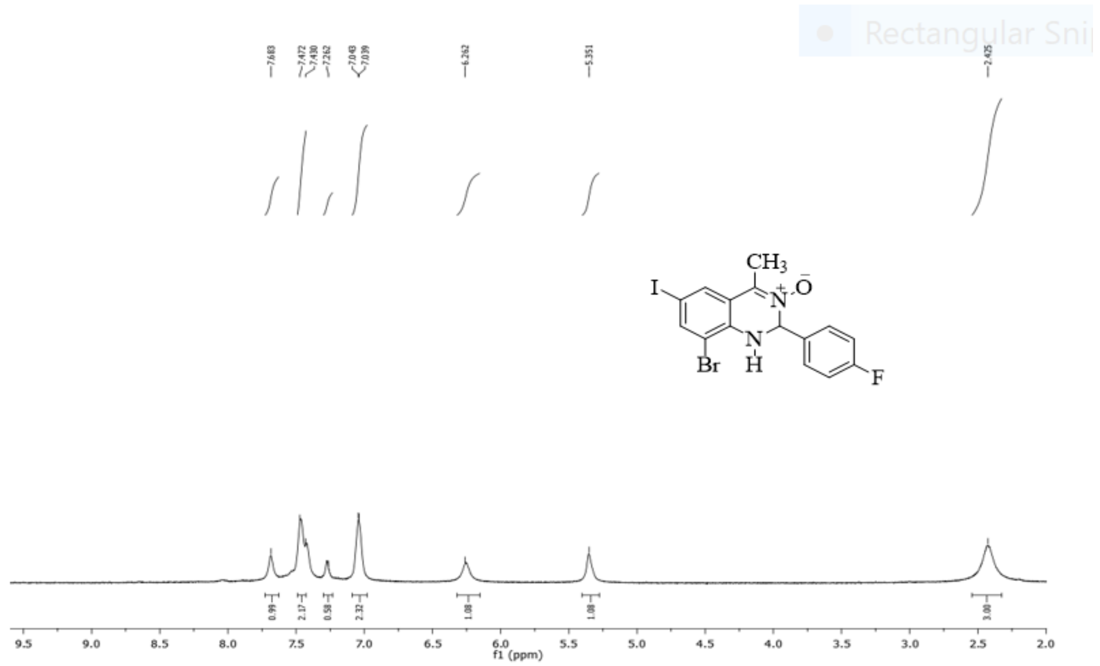

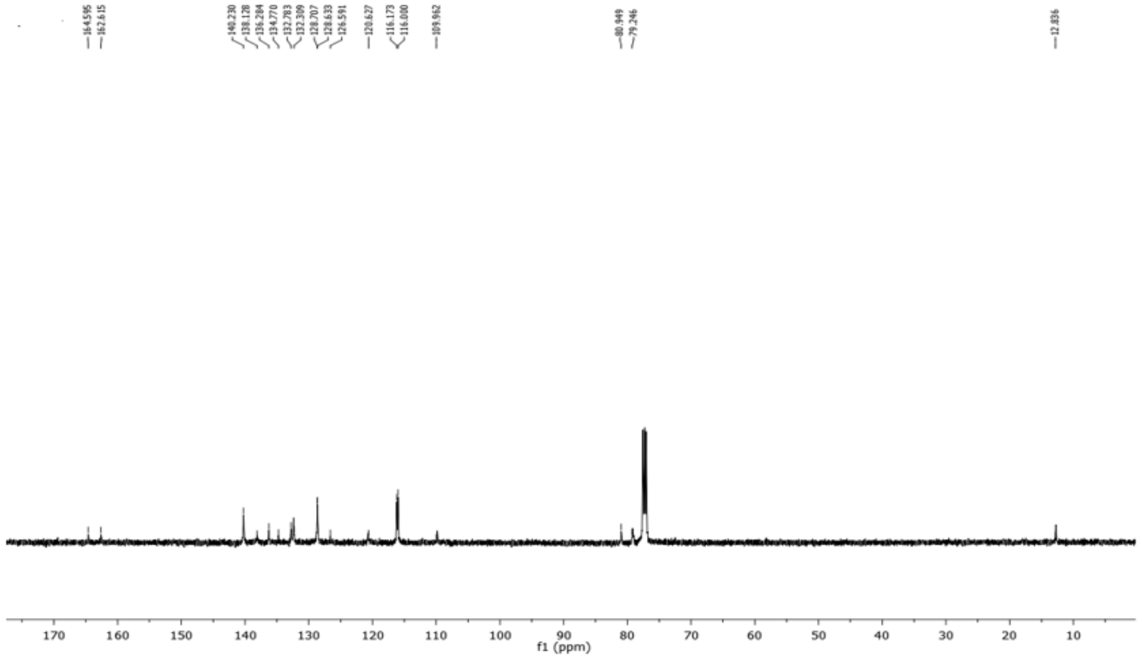

Fig. S1.18: ^{1}H- and ^{13}C-NMR spectra of **3n** in $CDCl_3$ at 500 MHz and 125 MHz, respectively.

8-Bromo-2-(4-fluorophenyl)-6-iodo-4-methyl-1,2-dihydroquinazoline 3-oxide (**3n**)

Yellow solid (0.30 g, 66%), R_f (5% EtOAc–toluene) 0.64, mp. 125–127 °C; ν_{max} (ATR) 861, 1095, 1187, 1231, 1487, 1591, 1669, 3057, 3278 cm^{-1}; ^{1}H-NMR (500 MHz, $CDCl_3$): δ 2.43 (s, 3H, CH_3), 5.35 (s, 1H, H-2), 6.26 (s, 1H, NH), 7.03 (t, J = 2.0 Hz, 2H, H-3',5'), 7.26 (s, 1H, H-5), 7.43 (2H, d, J = 21.0 Hz, 2H, H-2',6'), 7.68 (s, 1H, H-7); ^{13}C-NMR (125 MHz, $CDCl_3$) δ 12.8, 79.2, 80.9, 109.9, 116.0 (d, $^{2}J_{CF}$ = 21.6 Hz), 120.6, 126.6, 128.6 (d, $^{3}J_{CF}$ = 9.0 Hz), 132.3, 132.7 (d, $^{4}J_{CF}$ = 2.8 Hz), 134.8, 136.3, 138.1, 140.2, 162.6 (d, $^{1}J_{CF}$ = 247.5); HRMS (ES): MH^{+}, calcd for $C_{15}H_{12}N_2OBrFI$: 460.9083; found 460.9149.

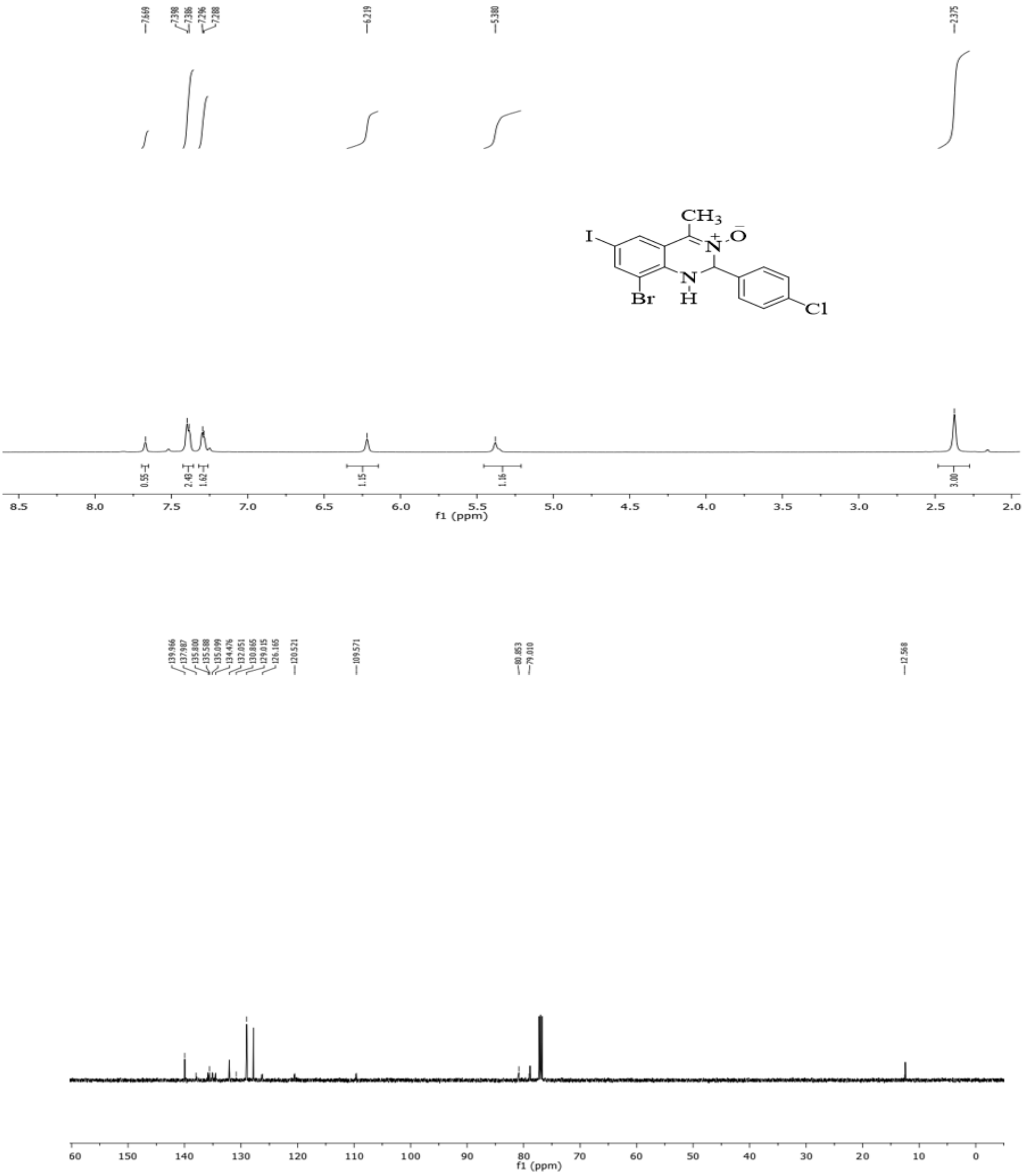

Fig. S1.19: ^{1}H- and ^{13}C-NMR spectra of **3o** in $CDCl_3$ at 500 MHz and 125 MHz, respectively.

8-Bromo-2-(4-chlorophenyl)-6-iodo-4-methyl-1,2-dihydroquinazoline 3-oxide (**3o**)

Yellow solid (0.49 g, 98%), R_f (5% EtOAc–toluene) 0.65, mp. 140–142 °C; ν_{max} (ATR) 856, 1082, 1184, 1234, 1446, 1485, 1589, 2920, 3250 cm^{-1}; ^{1}H-NMR (500 MHz, $CDCl_3$): δ 2.38 (s, 3H, CH_3), 5.38 (s, 1H, H-2), 6.22 (s, 1H, NH), 7.29 (d, *J* = 4.0 Hz, 2H, H-2′,6′), 7.39 (d, *J* = 6.0 Hz, 3H, H-3′,5′ and H-5), 7.69 (s, 1H, H-7); ^{13}CNMR (125 MHz, $CDCl_2$): δ 12.6, 79.0, 80.8, 109.6, 120.5, 126.2, 129.0, 130.8, 132.1, 134.5, 135.1, 135.6, 135.8, 137.9, 139.9; HRMS (ES): MH^{+}, calcd for $C_{15}H_{12}N_2OBrClI$: 476.8788; found 476.8388.

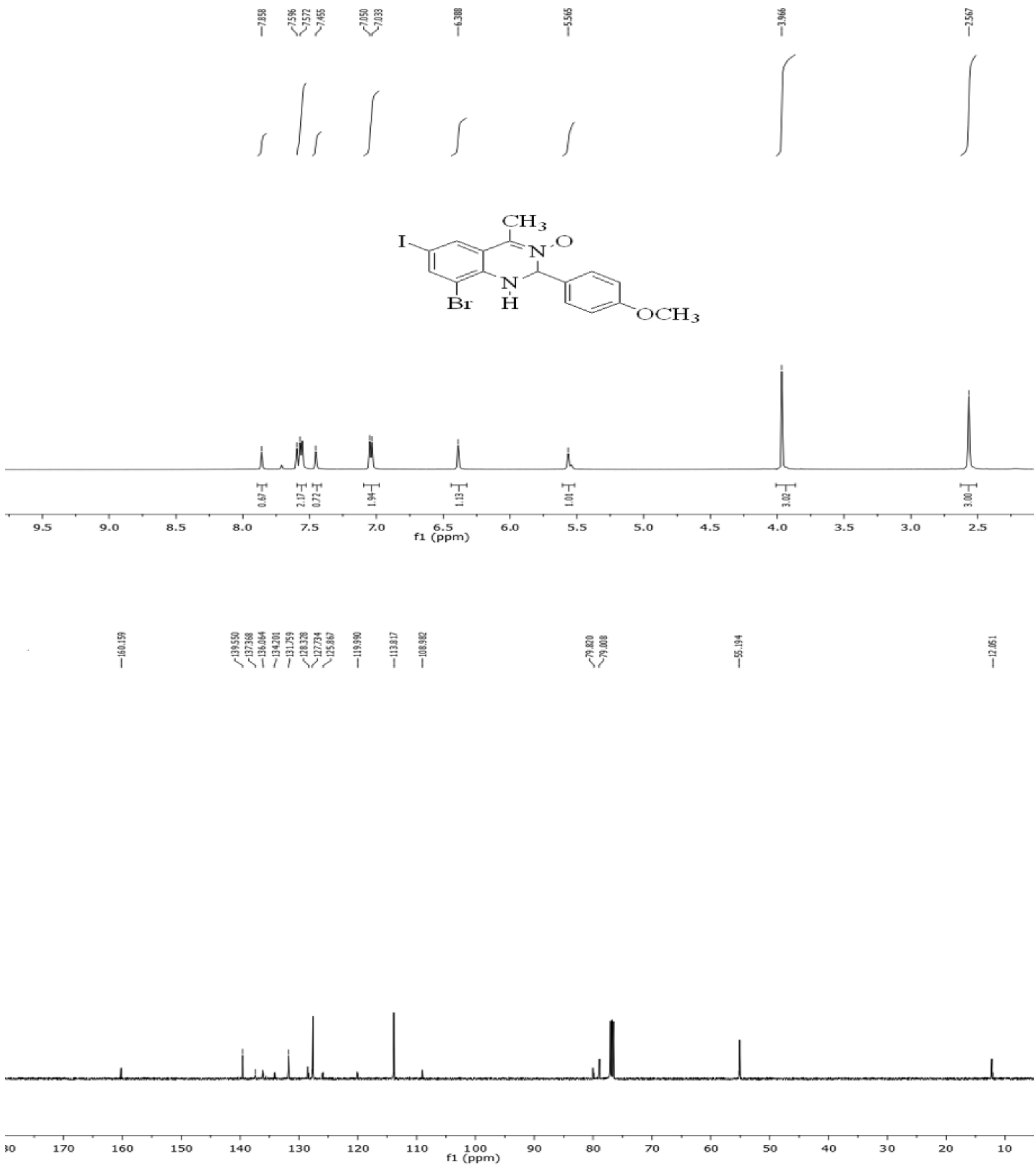

Fig. S1.20: ^{1}H- and ^{13}C-NMR spectra of **3p** in $CDCl_3$ at 500 MHz and 125 MHz, respectively.

8-bromo-6-iodo-2-(4-methoxyphenyl)-4-methyl-1,2-dihydroquinazoline 3-oxide (**3p**)

Yellow solid (0.47 g, 94%), R_f (5% EtOAc–toluene) 0.60, mp. 173–174 °C; ν_{max} (ATR) 856, 1082, 1184, 1234, 1446, 1485, 1589, 2920, 3250 cm^{-1}; ^{1}H-NMR (500 MHz, $CDCl_3$): δ 2.57 (s, 3H, CH_3), 3.97 (s, 3H, OCH_3), 5.57 (s, 1H, H-2), 6.39 (s, 1H, NH), 7.04 (d, J = 10 Hz, 2H, H-3′,5′), 7.46 (s, 1H, H-5), 7.58 (d, J = 12 Hz, 2H, H-2′,6′), 7.86 (s, 1H, H-7); ^{13}C-NMR (125 MHz, $CDCl_2$): δ 12.1, 55.2, 79.0, 79.8, 108.9, 113.8, 119.9, 125.9, 127.7, 128.3, 131.8, 134.2, 136.1, 137.4, 139.6, 160.2; HRMS (ES): MH$^+$, calcd for $C_{16}H_{15}N_2O_2BrI$: 472.9283; found 472.9385.

Table S1: X-ray analysis, crystal data and structure refinement for **3i**

Data collection and refinement of **3i**

Intensity data was determined on a Bruker Venture D8 Photon CMOS diffractometer with graphite-monochromated Mo$K\alpha_1$ (l = 0.71073 Å) radiation at 173(2) K using an Oxford Cryostream 600 cooler. Data reduction, empirical absorption corrections and space group assignments were carried out using the program SAINT+ version 6.02, SADABS and XPREP, respectively [3]. The structure was solved in the WinGX [4] Suite of programs, using intrinsic phasing through SHELXT [5] and refined using full-matrix least-squares/difference Fourier techniques on F^2 using SHELXL-2017 [6]. All C-bound hydrogen atoms were placed at idealized positions and refined as riding atoms with isotropic parameters 1.2 or 1.5 times those of their parent atoms. The *N*-bound hydrogen atom was located in the difference Fourier Map and fractional coordinates and isotropic displacement parameter refined freely. Diagrams and publication material were generated using ORTEP-3 [4] and PLATON [7].

Crystal data and structure refinement for **3i**

	**2a**
CCDC	2266298
Empirical formula	$C_{15}H_{12}BrIN_2O$
Formula weight	443.08
Crystal system	Monoclinic
Space group	*P*21/n
a, b, c (Å)	7.5889(3), 8.3516(3), 9.0732(4)
β (°)	90.312(5)
Volume (Å^3)	1540.4(3)
Z	4
ϱ_{calc} (*g/cm*^{3})	1.911
μ (mm^{-1})	4.669
F(000)	848
Crystal size (mm^3)	0.395 x 0.279 x 0.072
$\theta_{min}/\theta_{max}$ (°)	2.163/28.000
Index ranges	$-14 \leq h \leq 14$, $-10 \leq k \leq 10$, $-24 \leq l \leq 24$
Reflections collected	53714
Independent reflections	3717 [R(int) = 0.0659]
Data/restraints/parameters	3717 / 0 / 186
Goodness-of-fit on F^2	1.042
Final *R* indexes [$I \geq 2\sigma(I)$]	R_1 = 0.0240, wR2 = 0.0622
Final R indexes [all data]	R_1 = 0.0263, wR2 = 0.0637
Largest diff. peak/hole (*e.Å*$^{-3}$)	1.318 and -1.060

References

1. Ye, X.; Chen, Z.; Zhang, Z.; Fu, Y.; Deng, Z.; Peng, Y. An convenient approach to 2,4-disubstituted quinazoline-3-oxides using active MnO_2 as oxidant. *Canadian J. Chem*. **2019**, *97*, 682–689.
2. Mphahlele, M.J.; Magwaza, N.M.; Gildenhuys, S.; Setshedi, I.B. Synthesis, Synthesis, α-glucosidase inhibition, and antioxidant activity of the 7-carbo–substituted 5-bromo-3-methylindazoles. *Bioorg. Chem*. **2020**, *97*, 103702.
3. Bruker, APEX-3, SAINT+, Version 6.02 (Includes XPREP and SADABS), Bruker AXS Inc., Madison, Wisconsin, USA, **2016**.
4. Farrugia, L.J. WinGX and ORTEP for Windows an update. *J. Appl. Crystallogr*. **2012**, *245*, 849–854.
5. Sheldrick, G.M. SHELXT-Integrated space-group and crystal-structure determination. *Acta Cryst*. **2015**, *71*, 3–8.
6. Sheldrick, G.M. SHELXL-2017/1. *Acta Crystallogr*. **2015**, *C71*, 3–8.
7. Spek, A.L. Structure validation in chemical crystallography. *Acta Cryst*. **2009**, *D65*, 148-155.

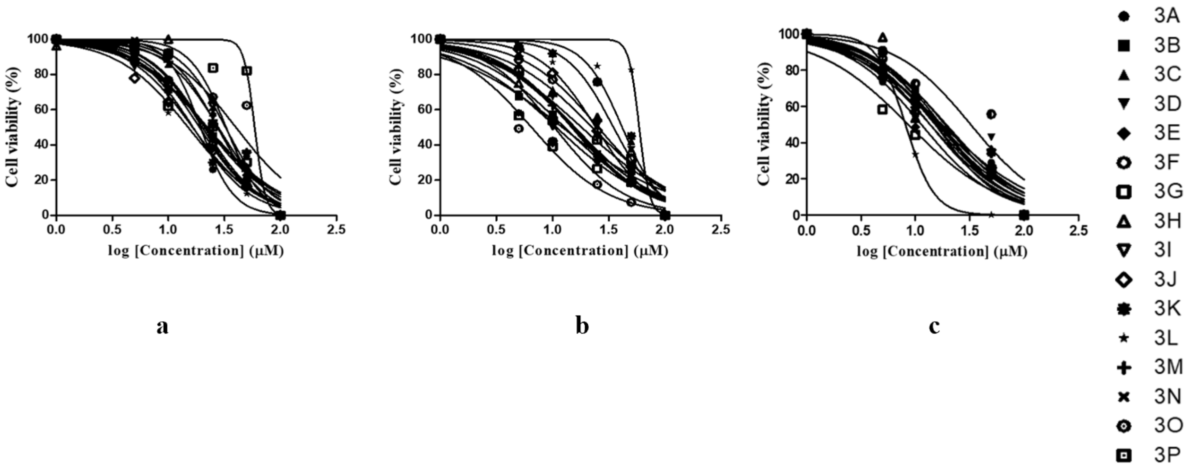

Fig. S2: Graphs showing dose-dependent effect on cell viability in MCF-7 (a), A549 (b) and HEK293-T (c).

Table S2: Cell viability percentage of compounds **3a–p** against MCF-7 cell line.

Conc.	3A	3B	3C	3D	3E	3F	3G	3H	3I	3J	3K	3L	3M	3N	3O	3P
**50**	19,6607	30,3061	37,2256	38,6394	17,2488	23,6814	33,9155	37,5083	18,1803	22,7375	38,1737	17,5102	17,6314	30,1896	23,0428	27,8817
**25**	22,3387	33,8157	41,6999	43,2136	19,7272	32,649	35,8117	41,2009	19,5775	30,7493	38,7891	35,1896	20,6088	31,7365	38,7891	43,212
**10**	23,187	35,1963	42,5981	44,0286	23,6194	44,9558	36,5769	43,1304	22,1058	40,413	39,6041	51,5919	20,9415	31,9528	39,6041	59,2339
**5**	26,1144	38,34	44,7938	46,8729	22,0559	62,4071	37,841	44,5775	22,006	53,8289	41,6001	55,5856	24,1517	33,2335	41,6001	70,8112
**1**	26,2641	39,7538	46,7898	47,0559	27,8443	69,8879	42,0991	45,0932	24,1184	60,354	42,1823	66,3742	25,998	34,8969	46,2908	72,4624

Table S3: Cell viability percentage of compounds **3a–p** against the A549 cell line.

Conc.	3A	3B	3C	3D	3E	3F	3G	3H	3I	3J	3K	3L	3M	3N	3O	3P
**50**	30,5771	28,8446	29,5534	39,174	30,3184	32,7708	33,0296	38,362	23,0847	29,7109	32,6808	45,4944	30,0709	30,3521	39,174	39,3879
**25**	41,1182	42,7495	41,782	44,944	40,2408	44,212	43,0757	56,0806	43,9195	42,6257	42,682	45,5732	42,2995	43,4807	44,944	42,5649
**10**	54,0443	56,1818	54,3481	46,8555	52,0419	61,143	50,6131	64,8667	61,098	57,7455	60,0068	60,333	61,413	62,0205	46,8555	43,6627
**5**	57,3068	60,8505	66,5879	66,5487	64,338	71,9204	56,2268	86,9389	69,8954	67,1729	73,6416	82,1465	68,6691	73,2703	66,5487	44,7771
**1**	66,8354	69,0066	73,4391	67,7876	74,9578	82,3715	72,7416	87,7714	84,0927	79,064	78,5578	89,6276	78,1978	79,9753	67,7876	45,0433

Table S4: Cell viability percentage of compounds **3a–p** against the HEK293-T cell line.

Conc.	3A	3B	3C	3D	3E	3F	3G	3H	3I	3J	3K	3L	3M	3N	3O	3P
**50**	19,0443	35,7618	23,5044	17,6402	20,0236	14,6903	17,0502	28,1437	26,8962	19,351	24,0118	19,7272	19,1032	20,4602	37,658	18,3835
**25**	55,0679	38,4731	34,1829	30,5988	31,941	36,7198	31,41	29,6574	29,8237	33,8879	31,5988	25,4809	41,9705	39,5988	44,2282	74,7257
**10**	57,8407	40,3194	50,3009	54,1003	40,0236	47,7522	41,062	30,7718	33,7658	54,4779	42,5133	59,208	51,1032	54,1003	44,3945	75,7994
**5**	63,351	41,3007	62,9381	60,0531	60,0118	55,2212	46,5251	32,5682	33,8656	72,767	55,3864	69,4229	75,0089	70,0531	44,5609	81,4277
**1**	69,3687	41,5502	64,6608	75,0561	69,7463	71,9646	63,41	33,3001	34,8969	67,5634	61,3333	80,3803	81,2389	75,0561	45,1763	83,4336

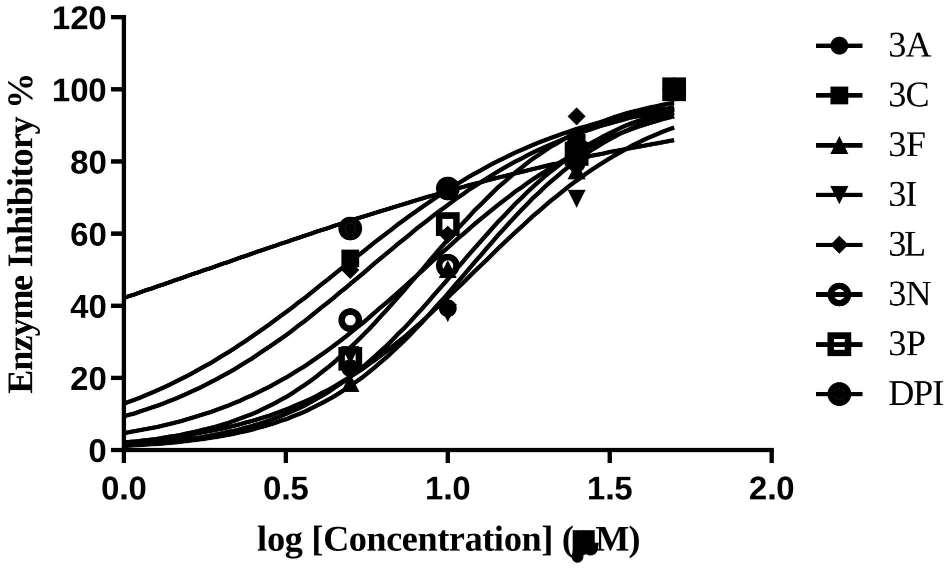

Fig. S3: Graphs of % **i**nhibition of SOD used to calculate IC_{50} values.

Table S5: Binding energies of **3a, 3c, 3f, 3i, 3l** and **3p** docked into α-glucosidase and α-amylase

	**Binding energy (kcal/mol)**	
**Compound**	**α-glucosidase**	**α-amylase**
**3a**	-19.5004	-9.4692
**3c**	-23.5707	-36.8609
**3f**	-27.3552	-32.4730
**3i**	-21.2141	-18.1673
**3l**	-22.5943	-37.7027
**3p**	-22.5534	-39.0532
Acarbose	-35.1447	-25.2018

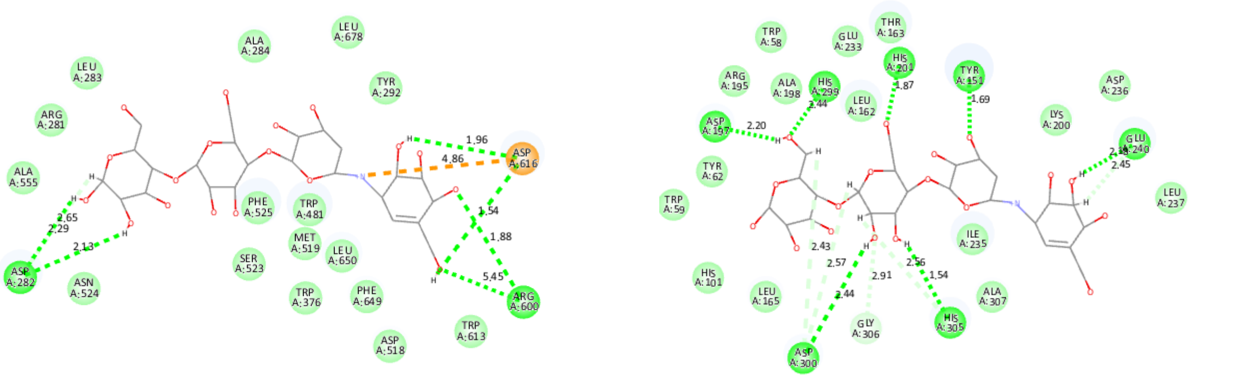

(a) (b)

Fig. S4: Docking poses of acarbose against α-glucosidase (a) and α-amylase (b). Interaction types are marked with dashed lines and are color-coded as follows: Bright green- conventional hydrogen bond; lighter green van der Waals interaction; very light green- carbon-hydrogen bond; orange is a salt bridge or attractive charge or pi-anion.
